# Bridging Size and Charge Effects of Mesoporous Silica Nanoparticles for Crossing the Blood–Brain Barrier

**DOI:** 10.3389/fchem.2022.931584

**Published:** 2022-06-27

**Authors:** Yi-Ping Chen, Chih-Ming Chou, Tsu-Yuan Chang, Hao Ting, Julien Dembélé, You-Tai Chu, Tsang-Pai Liu, Chun A. Changou, Chien-Wei Liu, Chien-Tsu Chen

**Affiliations:** ^1^ Graduate Institute of Nanomedicine and Medical Engineering, College of Biomedical Engineering, Taipei Medical University, Taipei, Taiwan; ^2^ International PhD Program in Biomedical Engineering, College of Biomedical Engineering, Taipei Medical University, Taipei, Taiwan; ^3^ Graduate Institute of Medical Sciences, College of Medicine, Taipei Medical University, Taipei, Taiwan; ^4^ Department of Biochemistry and Molecular Cell Biology, College of Medicine, Taipei Medical University, Taipei, Taiwan; ^5^ Graduate Institute of Biomedical Materials and Tissue Engineering, College of Biomedical Engineering, Taipei Medical University, Taipei, Taiwan; ^6^ Department of Surgery, Mackay Memorial Hospital, Taipei, Taiwan; ^7^ The PhD Program for Translational Medicine, College of Medical Science and Technology, Taipei Medical University, Taipei, Taiwan; ^8^ Department of Information Management, St. Mary’s Junior College of Medicine, Nursing and Management, Yilan, Taiwan

**Keywords:** blood–brain barrier, mesoporous silica nanoparticles, zebrafish, doxorubicin, protein corona

## Abstract

The blood–brain barrier (BBB) is a highly selective cellular barrier that tightly controls the microenvironment of the central nervous system to restrict the passage of substances, which is a primary challenge in delivering therapeutic drugs to treat brain diseases. This study aimed to develop simple surface modifications of mesoporous silica nanoparticles (MSNs) without external stimuli or receptor protein conjugation, which exhibited a critical surface charge and size allowing them to cross the BBB. A series of MSNs with various charges and two different sizes of 50 and 200 nm were synthesized, which showed a uniform mesoporous structure with various surface zeta potentials ranging from +42.3 to −51.6 mV. Confocal microscopic results showed that 50 nm of strongly negatively charged N4-RMSN_50_@PEG/THPMP (∼−40 mV) could be significantly observed outside blood vessels of the brain in *Tg*(*zfli1*:EGFP) transgenic zebrafish embryos superior to the other negatively charged MSNs. However, very few positively charged MSNs were found in the brain, indicating that negatively charged MSNs could successfully penetrate the BBB. The data were confirmed by high-resolution images of 3D deconvoluted confocal microscopy and two-photon microscopy and zebrafish brain tissue sections. In addition, while increasing the size to 200 nm but maintaining the similar negative charge (∼40 mV), MSNs could not be detected in the brain of zebrafish, suggesting that transport across the BBB based on MSNs occurred in charge- and size-dependent manners. No obvious cytotoxicity was observed in the CTX-TNA2 astrocyte cell line and U87-MG glioma cell line treated with MSNs. After doxorubicin (Dox) loading, N4-RMSN_50_@PEG/THPMP/Dox enabled drug delivery and pH-responsive release. The toxicity assay showed that N4-RMSN_50_@PEG/THPMP could reduce Dox release, resulting in the increase of the survival rate in zebrafish. Flow cytometry demonstrated N4-RMSN_50_@PEG/THPMP had few cellular uptakes. Protein corona analysis revealed three transporter proteins, such as afamin, apolipoprotein E, and basigin, could contribute to BBB penetration, validating the possible mechanism of N4-RMSN_50_@PEG/THPMP crossing the BBB. With this simple approach, MSNs with critical negative charge and size could overcome the BBB-limiting characteristics of therapeutic drug molecules; furthermore, their use may also cause drug sustained-release in the brain, decreasing peripheral toxicity.

## Introduction

The blood–brain barrier (BBB) serves as a physiological checkpoint to allow the passage of only certain molecules from the bloodstream into the brain. Indeed, the BBB is a continuous nonfenestrated endothelium in the brain composed of capillary endothelial cells, which are closely interconnected by tight junctions and surrounded by pericytes, astrocytes, basement membranes, and neurons (Daneman and Prat, 2015). The BBB routinely maintains central nervous system homeostasis by strictly regulating the exchange of extracellular ions and molecules as well as protecting neural tissues from injury from toxins and pathogens (Abbott et al., 2010; Moura et al., 2017). This defense checkpoint of the BBB is strictly selective in preventing access by most therapeutic molecules to the brain. Even though the transport of substances across the BBB depends on the properties of molecules, such as a small molecular weight and high lipophilicity, it was reported that almost all macromolecular neurotherapeutics and more than 98% of small-molecule drugs are unable to cross the BBB (Pardridge, 2005; Abbott et al., 2010; Pardridge, 2010). Hence, there is an urgent need to develop a drug delivery system to effectively transport therapeutic molecules into the brain.

There are several current mechanisms contributing to BBB transport technology, including passive diffusion, solute carriers (SLCs), and receptor-mediated and adsorptive-mediated transcytosis (Abbott et al., 2010; Chow and Gu, 2015). For example, receptor-mediated transcytosis, such as transferrin receptor (TfR), which is abundantly expressed by brain endothelial cells, has been widely utilized as a major pathway allowing substances to cross the BBB (Li et al., 2016; Choudhury et al., 2018). Recently, nanocarriers have received increasing attention in drug delivery and have shown several advantages, such as a high payload of drugs, controllable drug release, excellent targeting specificity, and enhancement of cellular uptake (Lee and Yeo, 2015; Choudhury et al., 2018; Cheng et al., 2019). New strategies that exploit receptor-mediated transcytosis combined with nanomaterials have enabled greater efficiency of drug delivery to the brain (Pulgar, 2019). In general, enhancing BBB drug transport using nanocarriers is performed through some protein–ligand modifications on the surface of nanoparticles (NPs) that improve the affinity to bind with receptors of endothelial cells in the brain, thus facilitating passage across the BBB. Proteins–ligands recruited for NPs surface modifications, such as transferrin, albumin, apolipoproteins, and lactoferrin, are widely used for nanocarrier engineering (Ku et al., 2010; Zhang et al., 2012; Cui et al., 2013; Hanada et al., 2014). However, the transport ability of receptor-mediated transcytosis may be less effective during disease progression, such as neuroinflammation, because expression levels of protein–ligand receptors could change (Schenk and De Vries, 2016). In addition, engineered nanocarriers with massive proteins–ligands may affect NPs properties resulting in recognition by an immune response, clearance from the circulation, and a change in the drug-release behavior. Meanwhile, the stability and specific activity of proteins–ligands during chemical conjugation could also become lower by either interference from subsequent modifications of the cargo due to steric hindrance or sensitization to organic solvents due to chemical denaturation. Therefore, there is much room for researchers to design promising NPs based on controlling size and simple surface modification, but without complex proteins–ligands conjugation, enabling BBB penetration.

Mesoporous silica nanoparticles (MSNs) are biocompatible nanomaterials due to their beneficial properties of easy functionalization, a tunable pore size, high surface area, and so on (Chen et al., 2013; Wu et al., 2013; Lin et al., 2016); and some evidence have demonstrated that MSNs are capable of penetrating the BBB. Heggannavar et al. (2018) found that significant transport of doxorubicin (Dox)-loaded silica NPs in the BBB vascular endothelial cell model was dependent on the functionalization of targeted ligands by transferrin. Song et al. (2017)investigated MSN surfaces conjugated with a targeted protein, lactoferrin, which had better BBB permeability and cellular uptake than non-conjugated MSNs did. Those results suggested that surface functionalization of MSNs plays an important role in BBB transport. However, there are few reports on BBB transport, especially *in vivo* by using native and simple MSNs without complex surface functionalization. In this study, we attempted to develop a native MSN without external stimuli or receptor protein conjugation, thus performing drug delivery across the BBB.

In recent years, zebrafish (*Danio rerio*) has emerged as a premier vertebrate model organism for studies of high-throughput drug screening, genetic mechanisms of embryonic development, neurodegenerative diseases, and BBB permeability because of short generation times, ease of cultivation, rapid embryogenesis, and adaptability to high-throughput screening processes (Jeong et al., 2008; Lessman, 2011; Umans and Taylor, 2012). Currently, many models ranging from *in vitro* cell models to *in vivo* animal models have been developed to evaluate the BBB (Vernon et al., 2011; Geldenhuys et al., 2012; Wilhelm and Krizbai, 2014; Jackson et al., 2019). In a preclinical BBB study, the zebrafish were considered the most attractive model organism for screening active compound libraries, toxicity tests, and *in vivo* BBB drug permeability studies. This is primarily due to certain biological features, such as the structure and function of the BBB in zebrafish being similar to those of mammalian species (Fleming et al., 2013). In this study, 3-day post-fertilization (dpf) embryos from *Tg*(*zfli1*:EGFP) zebrafish, which express enhanced green fluorescent protein (EGFP) in blood vessels under the driving of the zebrafish *fli1* promoter (Lawson and Weinstein, 2002), were employed as an *in vivo* animal model to evaluate the BBB permeability.

**SCHEME 1:**
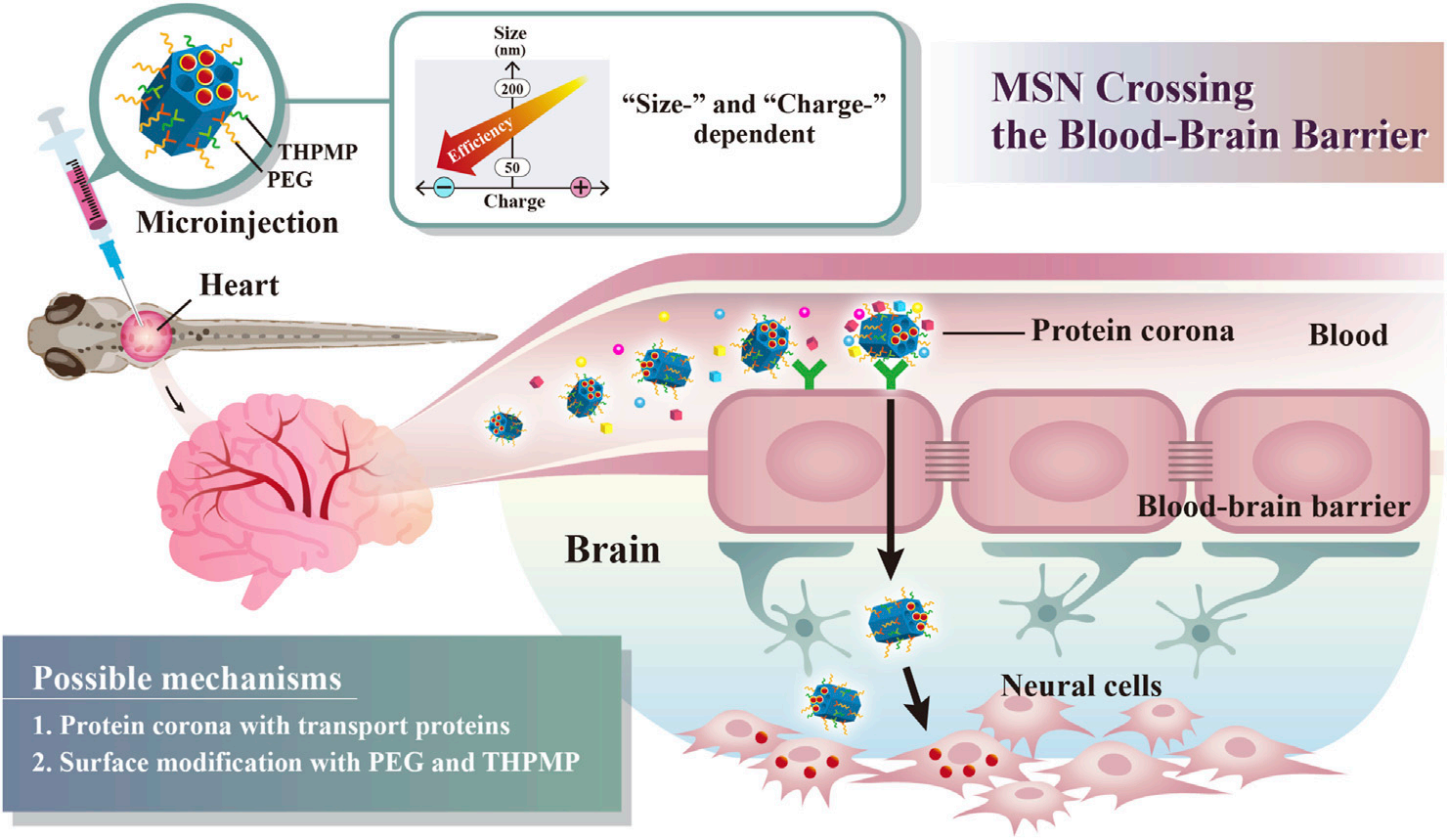
Schematic illustration of simple surface modifications of mesoporous silica nanoparticles (MSNs) without external stimuli or receptor protein conjugation, which exhibited a critical surface charge and size, enabling BBB crossing.

The aim of this study was to develop simple BBB-permeable MSNs by modifying different surface charges and sizes, which was capable of loading a drug (Scheme 1). We first synthesized four 50 nm rhodamine B isothiocyanate (RITC)-poly(ethylene glycol) (PEG)ylated MSNs with positively charged N-trimethoxysilylpropyl-N,N,N-trimethylammonium chloride (TMAC) and negatively charged (3-trihydroxysilyl)propylmethyl-phosphonate (THPMP), to produce weakly positively charged (P1-RMSN_50_@PEG/TMAC), strongly positively charged (P4-RMSN_50_@PEG/TMAC), weakly negatively charged (N1-RMSN_50_@PEG/THPMP), and strongly negatively charged (N4-RMSN_50_@PEG/THPMP) MSNs. Then, physical characterization including transmission electron microscopy (TEM), Brunauer–Emmett–Teller (BET), and dynamic light scattering (DLS) in water, PBS, serum-free and serum-containing media, and zeta potential measurements of MSNs were performed. The cytotoxicity was evaluated on CTX TNA2 (an astrocyte cell line) and U87 MG (a glioma cell line) cells. Thereafter, the biodistribution patterns of the MSNs in larval zebrafish and their brains were recorded by confocal microscopy and two-proton microscopy after microinjection into the pericardial cavity of *Tg*(*zfli1*:EGFP) transgenic zebrafish embryos. We found that N4-RMSN_50_@PEG/THPMP showed great penetration into the larval zebrafish brain. To further explore the critical negative charge effect of MSNs enabling penetration of the BBB, three different negatively charged MSNs (N2-RMSN_50_@PEG/THPMP, N3-RMSN_50_@PEG/THPMP, and N5-RMSN_50_@PEG/THPMP) were synthesized. Meanwhile, a larger strongly negatively charged MSN of 200 nm (N4-RMSN_200_@PEG/THPMP) was synthesized to investigate the size effect on BBB permeability. The results proved that a negative charge on MSN was essential for BBB penetration in larval zebrafish, and a small size was also decisive. To demonstrate the potential applications for brain diseases treatment, Dox-loaded MSN_50_@PEG/THPMP (MSN_50_@PEG/THPMP/Dox) showed pH-responsive and slow sustained-release behavior of drug, leading to the reduction cytotoxicity in U87 MG glioma cell line and the increase of the survival rate in zebrafish. The mechanism of MSNs transport to BBB was evaluated, focusing on the cellular uptake ability and protein corona analysis. Finally, we demonstrated that negatively charged MSNs without external stimuli or ligand/receptor protein conjugation could serve as nanocarriers to penetrate the BBB accompanied by drug release.

## Methods

### Chemicals and Reagents

Cetyltrimethylammonium bromide (CTAB, +99%), 3-aminopropyltrimethoxysilane (APTMS, 95%), tetraethyl orthosilicate (TEOS, 98%), and ammonium hydroxide solution (28–30 wt% NH_3_ in H_2_O) were obtained from Acros Organics. 2-(Methoxy(poly-ethyleneoxy)propyl) trimethoxysilane (PEG-silane, with a molecular weight of 460–590 g mol^−1^, 90%), N-trimethoxysilylpropyl-N,N,N-trimethylammonium chloride (TMAC, 50% in methanol), and (3-trihydroxysilyl)propylmethyl-phosphonate (THPMP, 42% in water) were supplied by Gelest. Rhodamine B isothiocyanate (RITC), 3-(4,5-dimethyl-2-thiazolyl)-2,5-dimethyl-2H-tetrazolium bromide (MTT) and doxorubicin hydrochloride (Dox) were purchased from Sigma-Aldrich. Absolute ethanol was purchased from Shimakyu Pure Chemicals. All cell culture reagents were obtained from Gibco. All reagents were used without further purification.

### Synthesis of PEGylated MSNs with Various Surface Charges

PEG-modified MSNs incorporating the RITC red fluorescence dye or not were synthesized with a diameter of either 50 (RMSN_50_@PEG) or 200 nm (RMSN_200_@PEG), by adjusting the ammonia concentration and temperature (Lu et al., 2009; Lin et al., 2011; Liu et al., 2015). Briefly, the RMSN@PEG obtained after the removal of surfactants possessed a weakly negatively charged surface and was designated N1-MSN@PEG. To obtain various RMSN@PEG MSNs with different surface charges, post-modifications were performed by negatively charged THPMP-silane and positively charged TMAC-silane, respectively, on the N1-RMSN@PEG to obtain N2 to N5 (with weak to strong negative charges) and P1 to P4 (with weak to strong positive charges)-RMSN@PEG MSNs. In more detail, for the syntheses of P1-RMSN_50_@PEG/TMAC and P4-RMSN_50_@PEG/TMAC, 200 mg of N1-RMSN_50_@PEG was dispersed in 100 ml EtOH and was stirred under reflux for 4 h with 0.36 and 3.6 mmol of TMAC-silane, respectively. For the syntheses of N2-, N3-, N4-, and N5-RMSN_50_@PEG/THPMP MSNs, after dispersing 200 mg of N1-RMSN_50_@PEG in a mixture of 80 ml H_2_O and 1 ml NH_4_OH, volumes of 10, 15, 20, and 25 ml of 0.44 M aqueous THPMP solution were respectively added. The mixture was continuously stirred at 40°C for 4 h. Also, to synthesize N4-RMSN_200_@PEG/THPMP MSNs, after dispersing 200 mg of N1-MSN_200_@PEG in a mixture of 80 ml H_2_O and 1 ml NH_4_OH, a volume of 20 ml of the THPMP solution was added under vigorous stirring at 40°C for 4 h. After that, the NPs were collected by centrifugation (11,000 rpm for 1 h) and subsequently washed with 95% ethanol. All NPs were dispersed in 99.5% ethanol and preserved until use.

### Characterization of NPs

TEM was used to observe the morphology and mesostructure of the NPs. The samples were prepared and dried by dropping them onto a carbon-coated copper grid and then observing them on a JEOL JSM-1200 EX II transmission electron microscope operated at 100 kV. Total surface areas and average pore diameters were determined by measuring the nitrogen adsorption–desorption isotherm on a Micrometerics ASAP 2010 apparatus and calculation by the BET and Barrett–Joyner–Halenda (BJH) methods. The hydrodynamic diameters of the NPs in water, PBS, serum-free, and culture medium supplemented with 10% fetal bovine serum (FBS) were assessed using DLS on a Malvern Zetasizer Nano ZS instrument. Zeta potential measurements were performed on a Malvern Zetasizer Nano ZS instrument by determining the electrophoretic mobility and applying Henry’s equation.

### Cell Culture

The human glioma U87-MG and rat astrocyte CTX TNA2 cell lines were cultured in minimum essential medium (MEM) and Dulbecco’s modified Eagle medium (DMEM) supplemented with 10% FBS, 100 units/ml penicillin, 100 μg/ml streptomycin, 1 mM sodium pyruvate, and 1 mM nonessential amino acids. These cell lines were incubated at 37°C in a humidified atmosphere of 5% CO_2_ and subcultured at 80% confluence.

### Cytotoxicity Assay

To measure the cytotoxicity induced by various types of RMSNs and Dox-loaded N4-MSN_50_@PEG/THPMP (N4-MSN_50_@PEG/THPMP/Dox) MSNs, an MTT ((3-(4,5-dimethyl-2-thiazolyl)-2,5-dimethyl-2H-tetrazolium bromide)) assay was conducted to determine the cell viability. The cells were seeded in 24-well plates at a density of 10^4^ cells/well, followed by subsequent treatment with different concentrations of various types of RMSNs (at 100 and 200 μg/ml) or free Dox (at 2–60 mg/ml) and an equivalent Dox dose of N4-MSN_50_@PEG/THPMP/Dox for 4 or 24 h in 10% serum-containing media. At the end of this treatment, 0.5 mg/ml MTT was added to each well for another 1 h. The supernatant was carefully removed, and MTT-formazan crystals were dissolved using DMSO. Finally, the absorbance was measured on a microplate reader (Thermo) at a wavelength at 570 nm.

### Zebrafish Maintenance

*Tg*(*zfli1*:EGFP) zebrafish (*D. rerio*) were maintained at the zebrafish core facility of Taipei Medical University in a water flow-through system at 28°C under a 14-h light/10-h dark cycle. Animal procedures were approved by the Taipei Medical University Institutional Animal Care and Utilization Committee (TMU-IACUC, Approval No. LAC-2018-0396). Embryos were staged according to the *Zebrafish Book*.

### Fluorescence Imaging Examination on Zebrafish Embryos

*Tg*(*zfli1*:EGFP) zebrafish embryos were injected with 200–300 nl (200–300 ng/injection) of various types of MSNs (1 mg/ml in H_2_O) into the pericardial cavity at 3 dpf. After being injected with various types of MSNs at 4 or 5 dpf, zebrafish embryos were anesthetized with 100 mg/L ethyl m-aminobenzoate (Tricaine, MS222) and embedded in 0.5% low-melting agarose. Fluorescent images were obtained by a confocal laser scanning microscope (TCS SP5, Leica) and two-photon microscopy (Olympus FVMPE-RS). The scanning time was according to the intensity of the fluorescence emitted. 3D digitally reconstructed images were computed by overlapping the sliding scans.

### Preparation of Dox-Loaded N4-MSN_50_@PEG/THPMP (N4-MSN_50_@PEG/THPMP/Dox)

N4-MSN_50_@PEG/THPMP MSNs (3 mg) without RITC conjugation were suspended in 400 μl of 0.1 M NaHCO_3_ for 2 h and then washed twice with double-distilled (dd) H_2_O. The N4-MSN_50_@PEG/THPMP MSNs were mixed with 0.25 mg of Dox in 400 μl of ddH_2_O. After stirring in dark at 4°C for 24 h, the mixture was centrifuged at 14,000 rpm and washed with ddH_2_O to remove any unloaded Dox. The amount of unloaded Dox was determined quantitatively by the fluorescence intensity of Dox at 480 nm (excitation) and 560 nm (emission) on a multi-well plate enzyme-linked immunosorbent assay (ELISA) reader (Bio-Rad). The Dox loading efficiency (%) and loading amount (μg Dox/mg NPs) were calculated by the following formulas:

$$\text{Loading efficiency }\left(\text{\%}\right)=\frac{Weight\;of\;Dox\;in\;N4-MSN50@PEG/THPMP/Dox}{Weight\;of\;total\;Dox}\times 100\%$$

and

$$\text{Loading amount}=\frac{Weight\;of\;Dox\;in\;N4-MSN50@PEG/THPMP/Dox}{Weight\;of\;N4-MSN50@PEG/THPMP/Dox}.$$

### *In Vitro* Dox Release Profile

To evaluate the release behavior of Dox *in vitro*, N4-MSN_50_@PEG/THPMP/Dox MSNs were placed into a dialysis membrane (MWCO:12–14 kDa) immersed in a phosphate-buffered saline (PBS) solution at different pH values (pH 5.5 and 7.4) and gently shaken at 37°C. At predetermined time intervals, 1 ml of a sample of the released Dox was collected, and the amount of Dox released was analyzed using a multi-well plate ELISA reader (Bio-Rad) with λex = 480 nm and λem = 560 nm (Dox).

### Evaluation of Cell Uptake

To evaluate cellular uptake of N4-RMSN_50_@PEG/THPMP, the human glioma U87-MG cells (1 × 10^5^) were seeded in a six-well plate. Following N4-RMSN_50_@PEG/THPMP treatment with indicated concentration (250, 500, and 750 μg/ml) in a 10% serum-containing medium for 24 h, the cells were washed with 1× PBS three times and then subjected to flow cytometry to quantify the efficiency of cell uptake by detecting the RITC dye fluorescence. For imaging, cell uptake and distribution of N4-RMSN_50_@PEG/THPMP with RITC-fluorescence signals were obtained from an inverted fluorescence microscope (Olympus IX71, Japan).

### Toxicity Assay on Zebrafish Embryos

Dechorionated embryos at 24 hpf were exposed individually to 2 ml of Dox (0.25 and 0.5 mg/ml), N4-MSN_50_@PEG/THPMP (4.5 and 9 mg/ml), and N4-MSN_50_@PEG/THPMP/Dox (4.5 and 9 mg/ml) at an equivalent dose of Dox in one well of a six-well chamber, and toxicity was evaluated by recording the embryo viability from 24 hpf to 96 hpf. Embryos were recorded and imaged in the plates by using Olympus IX70-FLA inverted fluorescence microscope (Olympus, Tokyo, Japan) at indicated time points.

### SDS-PAGE Analysis of Protein Corona

One mg of N4-RMSN_50_@PEG/THPMP was mixed with 300 μl of mouse plasma on an orbital shaker at 250 rpm for 30 min to mimic the dynamic *in vivo* blood flow condition. After that, the mixture was centrifuged at 15,570 × g for 30 min to separate the NPs-protein corona complex and then washed three times with PBS to remove the unbound and loosely bound corona proteins. The NPs-protein corona complex was added with Laemmli protein sample buffer, heated at 100°C for 10 min, and then loaded on 12% SDS-PAGE gel. Finally, the gel was stained with Coomassie blue and imaged.

### Protein Corona Composition Identification by Using LC-MS/MS

For LC-MS/MS analysis, the proteins from SDS-PAGE gel were sliced and subjected to in-gel trypsin digestion and extraction with 0.1% trifluoroacetic acid (TFA) in 50% acetonitrile (ACN). The proteins were analyzed on an Orbitrap Fusion Lumos Tribrid Mass Spectrophotometer (Thermo Fisher Scientific). The mass spectrometry proteomics data have been deposited to the ProteomeXchange consortium *via* the PRIDE partner repository with the dataset identifier PXD033783.

### Statistical Analysis

Each experiment was conducted at least three times and statistical analysis was performed using Student’s *t*-test. The data were processed by Origin and were expressed as mean ± standard deviation (SD). ***p < 0.001 was considered statistically significant.

## Results

### Synthesis and Characterization of Functionalized MSNs

Well-suspended and uniform RITC-conjugated MSNs (called RMSNs), with an average diameter of 50 nm, were prepared using diluted tetraethyl orthosilicate (TEOS) and a surfactant (CTAB) *via* an ammonia-catalyzed sol–gel process according to our previous reports (Lu et al., 2009; Lin et al., 2011; Liu et al., 2015). In order to avoid irreversible aggregation during synthesis, increase the dispersity and stability in biological milieu, prolong blood circulation *in vivo* through its hydrophilicity and steric repulsion effects (Aggarwal et al., 2009; Lowe et al., 2015), and retain good biocompatibility (Wu et al., 2011), PEG-silane was introduced on the surface of the as-synthesized MSNs by co-condensation. To track the biodistribution *in vivo*, the fluorescein RITC dye was incorporated into the framework of the MSNs. After removing the surfactant, the weakly negatively charged NPs were called N1-RMSN_50_@PEG. To obtain different types of RMSNs with various negative and positive surface charges, N1-RMSN_50_@PEG were respectively further post-modified with negatively charged THPMP-silane in a basic aqueous solution and with positively charged TMAC-silane in ethanol. Finally, four types of P/N-RMSN_50_@PEG with various zeta potentials were obtained: N1-RMSN_50_@PEG (weakly negatively charged), N4-RMSN_50_@PEG/THPMP (strongly negatively charged), P1-RMSN_50_@PEG/TMAC (weakly positively charged), and P4-RMSN_50_@PEG/TMAC (strongly positively charged).

**FIGURE 1:**
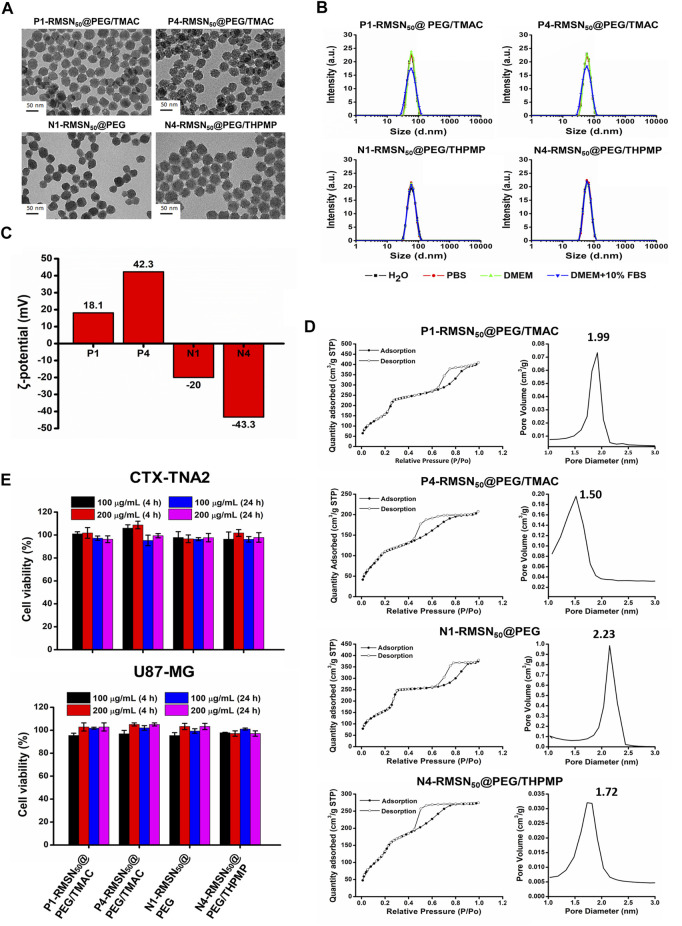
Physical characterizations and cytotoxicity of four types of RITC-conjugated mesoporous silica nanoparticles (RMSNs). **(A)** TEM images. Scale bar = 50 nm. **(B)** DLS, **(C)** zeta potential, and **(D)** nitrogen adsorption/desorption isotherms and corresponding pore size distribution plot of various types of RMSNs. **(E)** Cytotoxicity assay. CTX-TNA2 and U87-MG cells were cultured with different concentrations (100 and 200 μg/ml) of various types of RMSNs for 4 and 24 h in 10% serum-containing medium. Cell viability was detected using an MTT assay.

As shown in Figure 1A, TEM images indicated the various types of RMSNs with a size of around 50 nm, uniform morphology, and well-ordered pore structure. The hydrodynamic size of the NPs was measured by DLS (Figure 1B and Supplementary Table S1). The results revealed that all of the NPs were well dispersed in different solutions, and their Z-average hydrodynamic diameters were around 50 nm, which is in accordance with our previous results (Liu et al., 2015). Zeta potentials were in the range of −43.3 to +42.3 mV, confirming the presence of positively and negatively functional groups (Figure 1C). The N1-RMSN_50_@PEG was negatively charged with a zeta potential of −20 mV. After modification with negatively charged THPMP (N4-RMSN_50_@PEG/THPMP), the zeta potential decreased to −43.3 mV. After surface post-modification with TMAC, a positively charged group, the zeta potential of P1-RMSN_50_@PEG/TMAC dramatically increased to +18.1 mV. After further conjugation with additional TMAC, P4-RMSN_50_@PEG/TMAC exhibited a strongly positive surface charge (+42.3 mV). Nitrogen adsorption/desorption using the BET method exhibited type IV isotherms, consistent with a mesoporous structure (Figure 1D). As shown in Figure 1D, average pore diameters according to BJH calculation ranged 1.99–1.50 (RMSN_50_@PEG/TMAC) and 2.23–1.72 (RMSN_50_@PEG/THPMP). The decrease in the pore size was due to functional groups filling in the pores of the RMSNs.

Previous reports suggested that engineered NPs are not only capable of crossing the BBB but also induce serious cytotoxicity to the brain, which was associated with surface properties of the NPs, such as the size and surface charge (Trickler et al., 2010; Pandey et al., 2015; Liu et al., 2017). We then evaluated the *in vitro* cytotoxicity of various types of RMSNs on two brain-related cell lines: the CTX-TNA2 astrocyte cell line and U87-MG glioma cell line. Cell viability as examined by the MTT assay showed no significant change in either CTX-TNA2 or U87-MG cells treated with different concentrations (100 or 200 μg/ml) of various types of RMSNs for 4 or 24 h in 10% serum-containing media condition (Figure 1E). Astrocyte cells play an important role in determining the BBB integrity (Moura et al., 2017). Results showed no obvious cytotoxicity toward astrocytes or glioma cells, demonstrating that these differently charged RMSNs of 50 nm in diameter were not harmful and exhibited good biocompatibility.

### Effect of Surface Functionalization of MSNs on BBB Penetration in Zebrafish Embryos

**FIGURE 2:**
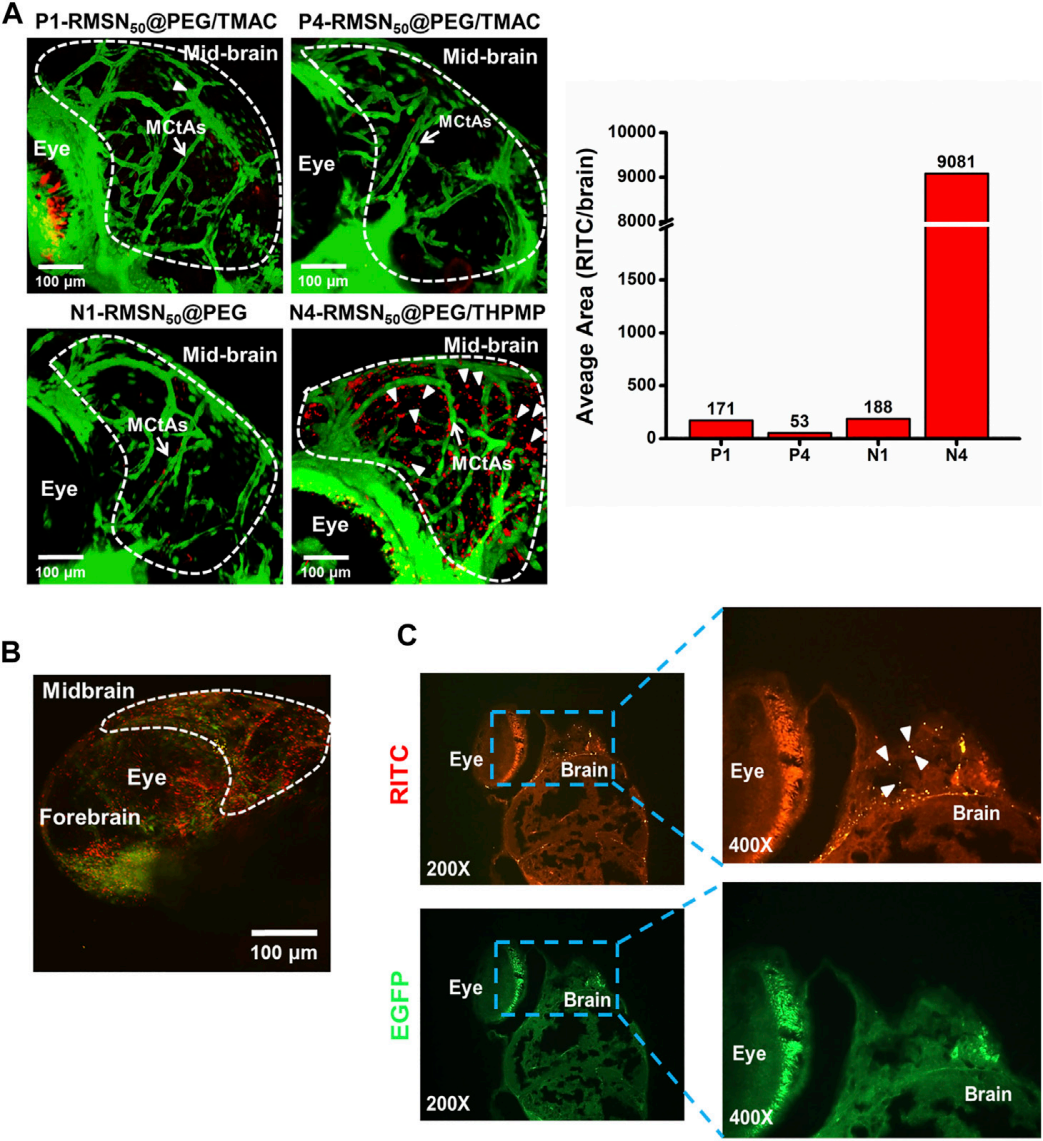
Biodistribution in the brain of four types of RITC-conjugated mesoporous silica nanoparticles (RMSNs) after a pericardial cavity injection in *Tg*(*zfli1*:EGFP transgenic zebrafish (200–300 ng/embryo) at 4 days post-fertilization (dpf). **(A)** Confocal microscopy imaged the distributions of various types of RMSNs in the zebrafish larval brain. The white arrow indicates mesencephalic central arteries (MCtAs). Arrowheads indicate that RMSNs (red) were outside the brain’s blood vessels (green). **(B)** Two-photon microscopic images and **(C)** fluorescence microscopic analysis of zebrafish embryo brains after injection with N4-RMSN_50_@PEG/THPMP. Red (RITC) fluorescence and green (GFP) fluorescence, respectively, represent RMSNs and blood vessels. Arrowheads indicated RMSNs (red).

To study the biodistribution of RMSNs, an *in vivo Tg*(*zfli1*:EGFP) transgenic zebrafish model was used. After injection of various types of RMSNs (200–300 ng/injection) into the pericardial cavity of 3-dpf zebrafish embryo, the biodistributions of RMSNs were observed by fluorescence microscopy 24 h after the injection. The results indicated that the biodistribution in zebrafish embryos treated with positively charged P1- and P4-RMSN_50_@PEG/TMAC resulted in a similar pattern, in which red fluorescence was detected in the epithelial cells, the yolk, dorsal aorta, and caudal aorta (Supplementary Figure S1); in contrast, the distribution of negatively charged N1 or N4-RMSN_50_@PEG/THPMP was observed to be in the circulation (dorsal aorta and cardinal vein), and weakly in epithelial cells and yolk; however, significantly strong red fluorescence was localized in the brain of embryos treated with N4-RMSN_50_@PEG/THPMP. To further analyze the distributions of various types of RMSNs in the brain, confocal microscopic images were examined (Figure 2A). In the brain of larval zebrafish treated with strongly negatively charged MSNs (N4-RMSN_50_@PEG/THPMP), the image showed that brilliant red fluorescence was observed outside blood vessels (green), suggesting that this RMSN had a higher penetration ability to cross the BBB. However, RMSNs with a positive charge (P1 and P4-RMSN_50_@PEG/TMAC) or a weak negative charge (N1-RMSN_50_@PEG) were scantily present in the brain. 3D deconvoluted confocal fluorescence images also confirmed these results (Supplementary Videos S1, S2).

Meanwhile, two-photon microscopy was employed to display the N4-RMSN_50_@PEG/THPMP distribution in the brain of zebrafish with high-resolution images, clearly showing the red fluorescence outside blood vessels, which was consistent with results of confocal microscopy (Figure 2B). To further confirm the results in detail, analysis by fluorescence microscopy of zebrafish brain tissue section images was performed after treatment with N4-RMSN_50_@PEG/THPMP. Figure 2C presented the obvious localization of N4-RMSN_50_@PEG/THPMP in the brain. Therefore, we found that RMSNs with strong negative charges could successfully penetrate the BBB of zebrafish.

### Charge and Size Effects of MSNs on BBB Penetration

**FIGURE 3:**
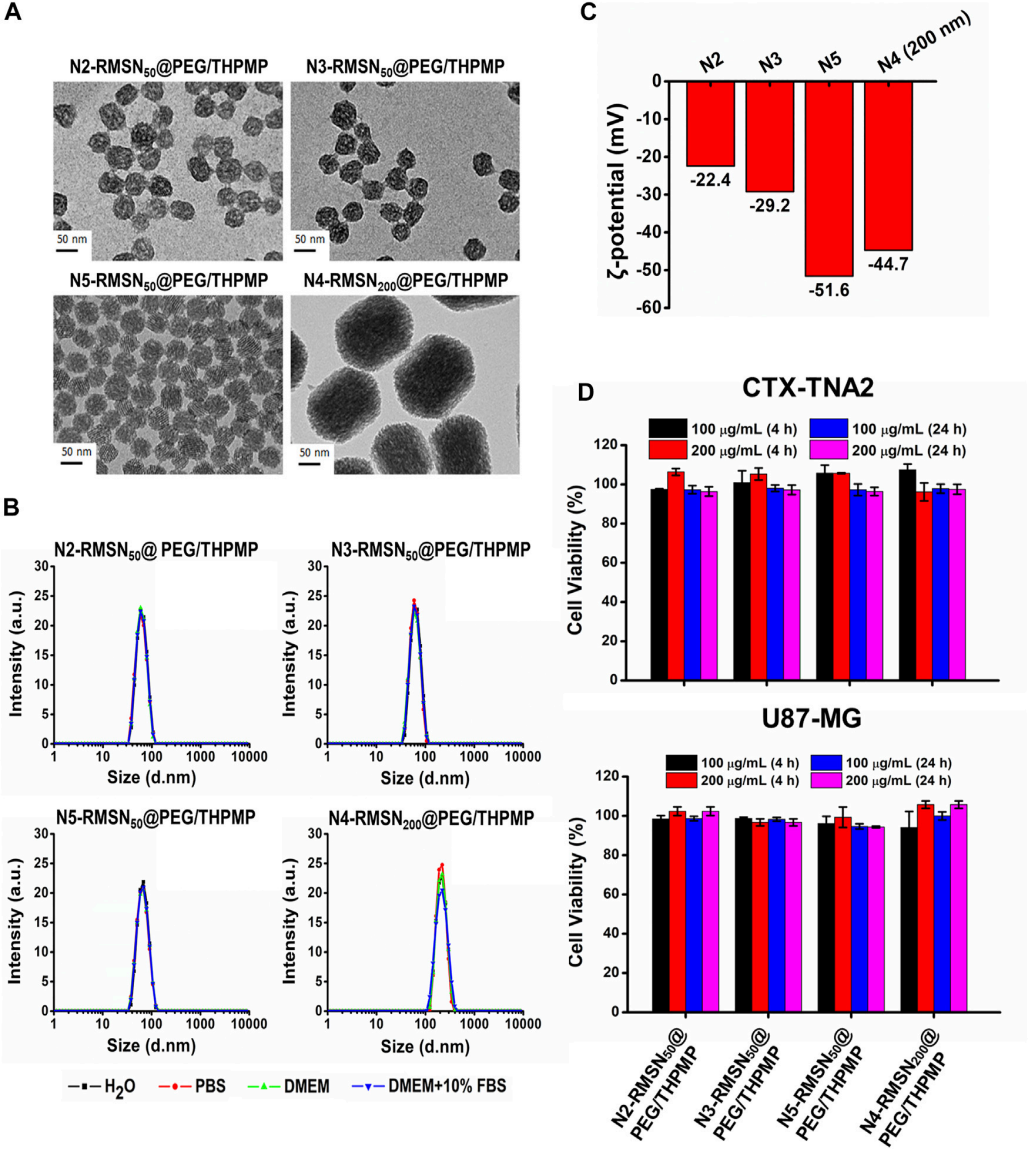
Physical characterizations and cytotoxicity of four different negatively charged RITC-conjugated mesoporous silica nanoparticles (RMSNs) with different sizes (50 and 200 nm). **(A)** TEM images. Scale bar = 50 nm. **(B)** DLS and **(C)** zeta potential measurements. **(D)** Cytotoxicity assay. CTX-TNA2 and U87-MG cells were cultured with different concentrations (100 and 200 μg/ml) of various types of RMSNs for 4 and 24 h under 10% serum-containing media condition. Cell viability was detected with an MTT assay.

Since a stronger negative charge is essential for the better ability to cross the BBB, the critical negative charge and particle size affecting BBB permeability were further explored. We subsequently synthesized a series of negatively charged RMSNs with a lower negative charge than N4-RMSN_50_@PEG/THPMP (N2-RMSN_50_@PEG/THPMP and N3-RMSN_50_@PEG/THPMP) and one with a higher negative charge (N5-RMSN_50_@PEG/THPMP). To investigate the size effect, strongly negative-charged MSNs with a diameter of 200 nm (N4-RMSN_200_@PEG/THPMP) were synthesized for further studies. All of the synthesis NPs showed a well-ordered mesoporous structure on TEM images (Figure 3A). DLS characterization (Figure 3B) revealed that all of the NPs were easily suspended in different solutions and they exhibited expected sizes of around 50 and 200 nm. Zeta potential measurements showed that the expected negative surface charge ranged from −22.4 to −51.6 mV, depending on the different ratios of THPMP functionalization (Figure 3B). It was noted that N4-RMSN_50_@PEG/THPMP (−43.3 mV) and N4-RMSN_200_ @PEG/THPMP (−44.7 mV) had similar zeta potentials to investigate the size effect on BBB transport. The MTT assay demonstrated that none of the NPs at concentrations of 100 and 200 μg/ml caused cytotoxicity in CTX-TNA2 or U87-MG cells following treatment for 4 and 24 h (Figure 3C).

**FIGURE 4:**
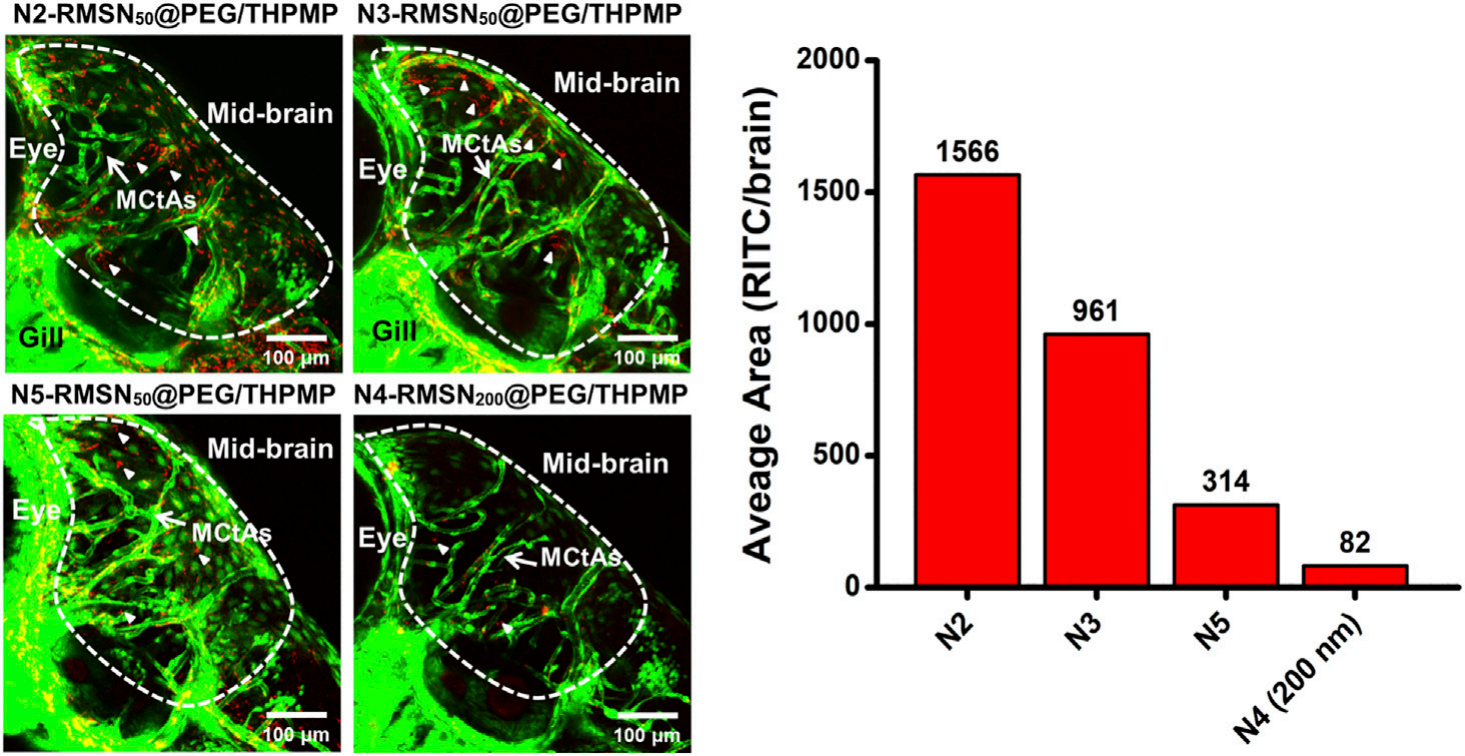
Confocal microscopic images of four different negatively charged RITC-conjugated mesoporous silica nanoparticles (RMSNs) with different sizes (50 and 200 nm) in zebrafish larval midbrain. White arrows indicate mesencephalic central arteries (MCtAs); arrowheads indicated red fluorescence (RITC) of nanoparticles outside the brain’s blood vessels (GFP, green).

These NPs were then injected into the pericardial cavity of *Tg*(*zfli1*:EGFP) transgenic zebrafish embryos (3 dpf) to verify their distribution in the larval zebrafish brain (Figure 4). Confocal microscopic images demonstrated that all three of N2-, N3-, and N5-RMSN_50_@PEG/THPMP were distinctly observed outside blood vessels in the larval brain (white arrows) in a similar pattern to that of N4-RMSN_50_@PEG/THPMP. However, the amount of N5-RMSN_50_@PEG/THPMP in the brain was estimated to be small compared to that of N4-RMSN_50_@PEG/THPMP. N4-RMSN_200_@PEG/THPMP was larger, had the same surface charge as N4-RMSN_50_@PEG/THPMP, and showed poor penetration into the larval zebrafish brain, because fewer red fluorescent particles were found (arrowheads) (Supplementary Video S3). These results demonstrated that penetration of RMSNs into the zebrafish brain occurred in a negative charge-dependent and size-dependent manner. It was also consistent with larger NPs having a poorer ability to cross the BBB (Liu et al., 2017; Song et al., 2017; Heggannavar et al., 2018). Therefore, MSNs having a charge between −20 and −40 mV and a size in 50 nm are critical for crossing the BBB.

### Dox Release and Delivery Based on MSNs *In Vitro* and *In Vivo*

**FIGURE 5:**
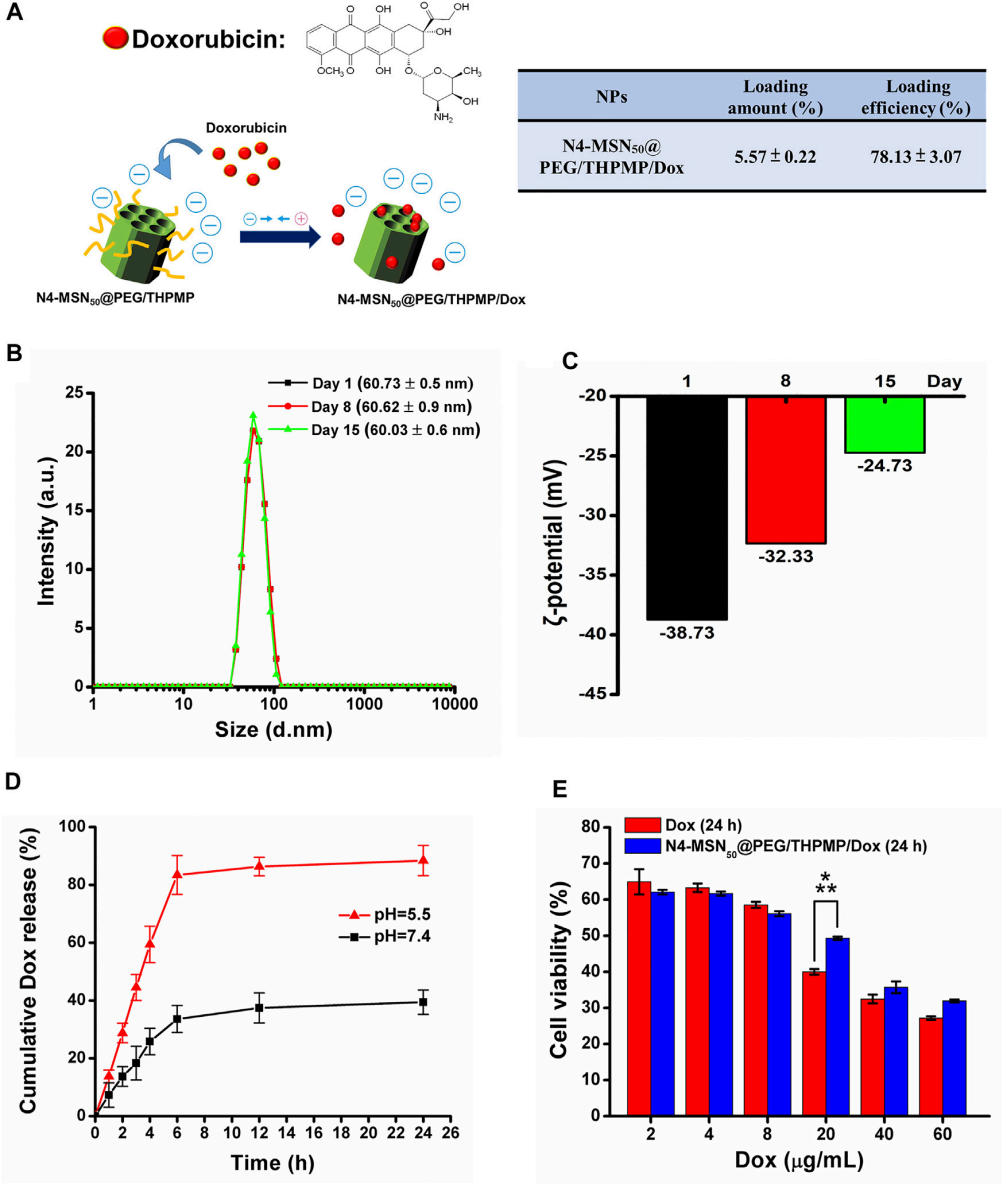
N4-MSN_50_@PEG/THPMP-mediated doxorubicin (Dox) delivery against the U87-MG cells and their stability. **(A)** Schematic representation of Dox loading strategy and the loading capacity**.** Stability assay of N4-MSN_50_@PEG/THPMP/Dox by using **(B)** hydrodynamic diameter distributions and **(C)** zeta potential measurements in water at day 1, 8, and 15. **(D)** *In vitro* release profiles of Dox in PBS buffer (pH 7.4 and 5.5) at a physiological temperature (37°C). **(E)** *In vitro* cytotoxicity of U87-MG cells treated with different concentrations of free Dox or Dox-loaded N4-MSN_50_@PEG/THPMP (equivalent dose of Dox) for 24 h ***p < 0.001.

To further estimate the possibility of using N4-RMSN_50_@PEG/THPMP as a drug carrier for brain delivery, Dox was used for drug loading. Dox also exhibits red fluorescence (Motlagh et al., 2016) so it is easy to trace its localization *in vivo* from MSN release after delivery. First, non-fluorescent N4-MSN_50_@PEG/THPMP MSNs were synthesized instead of N4-RMSN_50_@PEG/THPMP in order to avoid interference from RITC fluorescence. The loading strategy was based on electrostatic interactions between the negative charge of THPMP and the positive charge of Dox (Figure 5A). The loading efficiency and loading amount of N4-MSN_50_@PEG/THPMP/Dox were 78.13 ± 3.07% and 5.57 ± 0.22 wt%, respectively (Figure 5A). For medical applications, the stability of N4-MSN_50_@PEG/THPMP/Dox was then evaluated. After Dox loaded, the size of N4-MSN_50_@PEG/THPMP/Dox did not significantly change with around 60 nm in water according to DLS measurement (Figure 5B). Despite Dox loading, N4-MSN_50_@PEG/THPMP/Dox was also stable when maintained in water within 15 days because of no significant aggregation from DLS measurement. The results indicated that N4-MSN_50_@PEG/THPMP/Dox was suitable for *in vivo* experiment use. Zeta potential revealed that values of the charge of N4-MSN_50_@PEG/THPMP/Dox were around −39 mV at day 1, −32 mV at day 8, and−25 mV at day 15 (Figure 5C); it became more positive, but was still within the negative charge range of N2-RMSN_50_@PEG/THPMP to N4-RMSN_50_@PEG/THPMP, which was beneficial to BBB penetration. Then, *in vitro* release profile of Dox was determined by incubating N4-MSN_50_@PEG/THPMP/Dox in PBS buffer at pH 7.4 (a physiological environment) and pH 5.5 (an acidic environment) at 37°C as shown in Figure 5D. We found that more than 80% of the loaded Dox was released from N4-MSN_50_@PEG/THPMP/Dox incubated in PBS buffer at pH 5.5 within 6 h; in contrast, only about 30% of the loaded Dox was released at pH 7.0. This result indicates the cumulative time-dependent release and a massive release in an acidic condition (pH 5.5); thus it is possible to modulate slow sustained-release and pH-responsive release. With the benefits of sustained-release Dox, *in vitro* cytotoxicity of N4-MSN_50_@PEG/THPMP/Dox against human U87-MG glioma cells by using the MTT assay was examined. The cells were treated with free Dox (2–60 mg/ml) and equivalent Dox doses of N4-MSN_50_@PEG/THPMP/Dox for 24 h. As shown in Figure 5E, both free Dox and N4-MSN_50_@PEG/THPMP/Dox showed dose-dependent cytotoxic effects, indicating the effective release of Dox.

**FIGURE 6:**
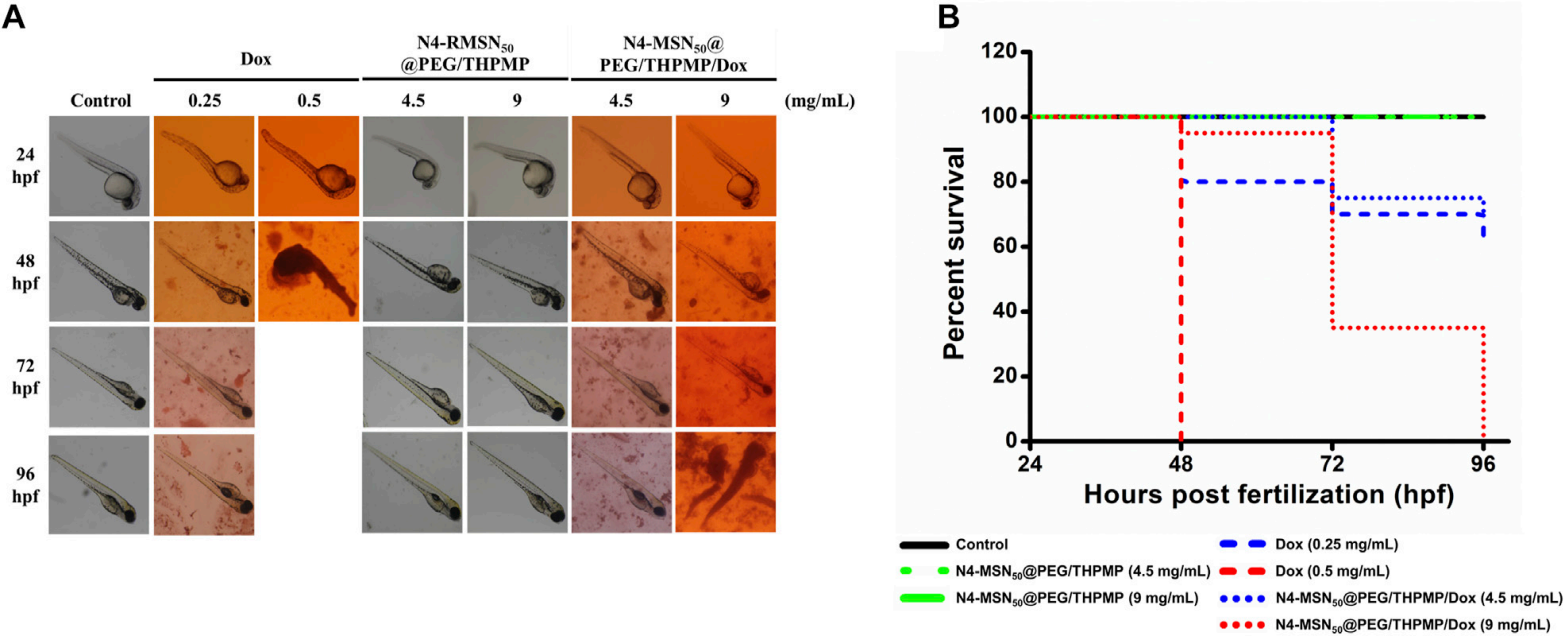
N4-RMSN_50_@PEG/THPMP decreased doxorubicin (Dox) induced toxicity and increased the survival rate. **(A)** Representative optical images of zebrafish at 24 hpf stage after exposure to Dox (0.25 and 0.5 mg/ml), N4-MSN_50_@PEG/THPMP (4.5 and 9 mg/ml), and N4-MSN_50_@PEG/THPMP/Dox (4.5 and 9 mg/ml) at an equivalent dose of Dox for 24, 48, 72, and 96 hpf. **(B)** Kaplan–Meier plots of overall survival. *N* = 20 (magnification ×50).

In addition to *in vitro* cytotoxicity study, the *in vivo* zebrafish model was employed to investigate whether N4-MSN_50_@PEG/THPMP/Dox could decrease Dox induced toxicity (Figure 6). There was no toxicity in the treatment of N4-MSN_50_@PEG/THPMP under 4.5 mg/ml and 9 mg/ml concentration. In contrast, zebrafish treated with Dox and N4-MSN_50_@PEG/THPMP/Dox showed time-dependent toxicity. On the 48 h post-incubation, 9 mg/ml of N4-MSN_50_@PEG/THPMP/Dox (0.5 mg/ml of Dox equivalent dose) treatment displayed 95% excellent survival rate superior to free Dox (0.5 mg/ml) with 0%. The Kaplan–Meier plots of overall survival was shown in Figure 6B and detailed subgroup and statistical analysis was depicted in Supplementary Table S3. The increased survival rate was due to the improvement of Dox toxicity by N4-MSN_50_@PEG/THPMP/Dox.

### Mechanism of Protein Corona and Surface Modification of MSNs Affecting the BBB Crossing

**FIGURE 7:**
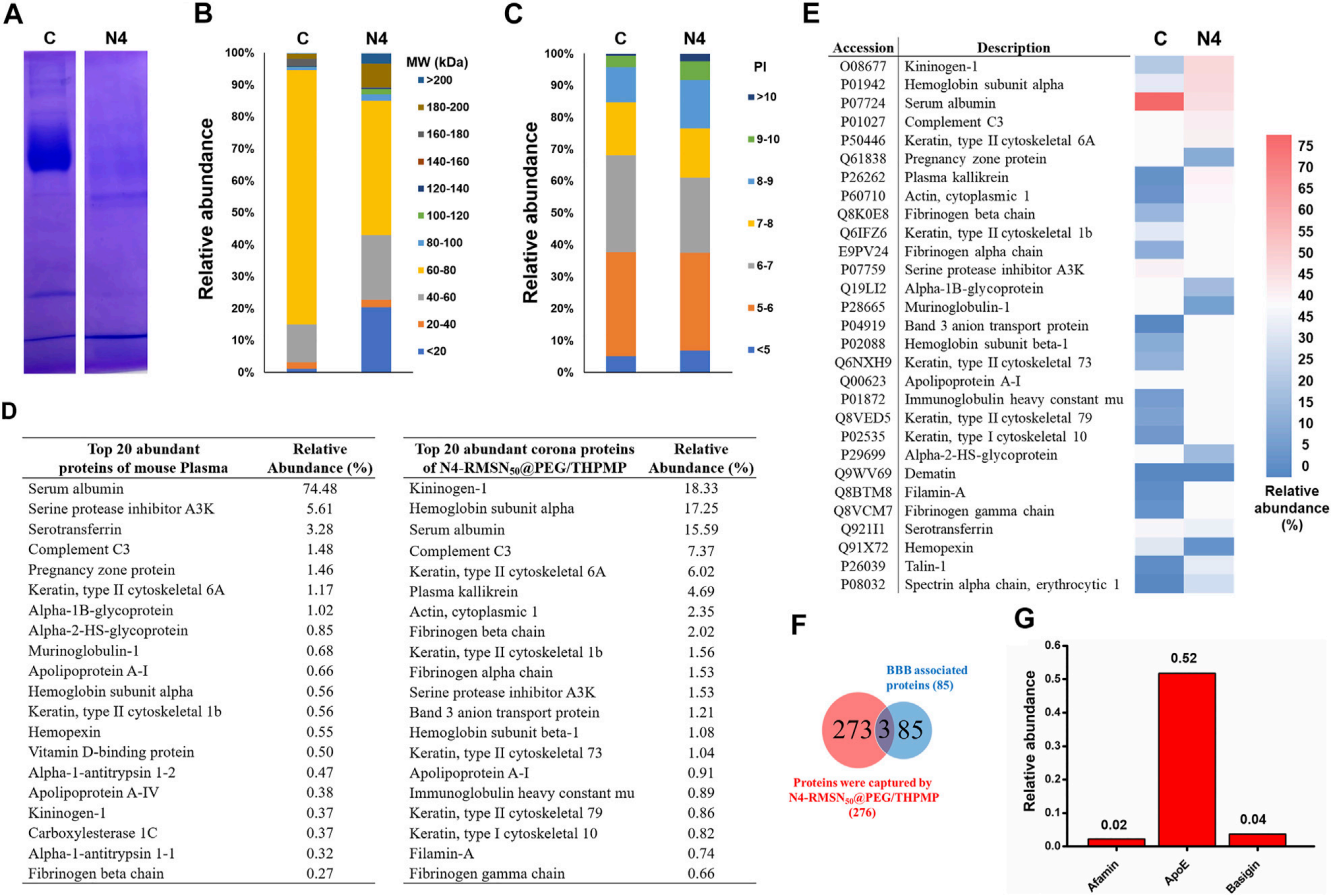
Comprehensive protein corona identification of N4-RMSN_50_@PEG/THPMP incubated with mouse serum by LC-Ms/MS. **(A)** SDS-PAGE gel of plasma (control) and corona proteins associated with N4-RMSN_50_@PEG/THPMP. **(B)** Classification of corona protein according to **(C)** molecular weight. and **(D)** isoelectric point. The most abundant proteins (top 20) are summarized. **(E)** Heatmap of the most abundant proteins. **(F)** Venn diagrams report the number of protein corona and the proteins related to BBB transport. **(G)** Relative abundance (vs mean abundance) of BBB transport associated proteins identified in the protein corona. C: control. N4: N4-RMSN_50_@PEG/THPMP.

Once NPs were administered into the physiological condition, several serum proteins could adsorb rapidly onto the NPs to form a protein layer (also called protein corona). The effect of protein corona surrounding the NPs depends on several parameters such as size, charge and other surface properties of NPs, which could contribute a great deal of influence in determining the NP’s fate. Hence, we explored the possible mechanism of the transport of N4-RMSN_50_@PEG/THPMP into the BBB by analyzing the interaction with mouse serum proteins. In this study, we used mouse serum as an alternative protein source for the protein corona analysis because of the insufficient availability of zebrafish blood serum for collection to perform the analysis. First, we investigated the *in vitro* protein corona pattern of N4-RMSN_50_@PEG/THPMP incubated in mouse serum and demonstrated that several protein bands retained by SDS-PAGE analysis (Figure 7A). The protein bands were extracted and then characterized by LC-MS/MS, a total of 273 proteins were identified which could interact with N4-RMSN_50_@PEG/THPMP. Compared to control plasma, N4-RMSN50@PEG/THPMP captured proteins in difference with various content of molecular weights (MW) (Figure 7B). The abundance pattern indicated that some lower MW (below 20 KD and 40–60 KD) and some higher MW (above 80 KD) proteins were highly enriched after captured. Moreover, the isoelectric point analysis of captured proteins indicated that the content of higher isoelectric point (above eight and higher) proteins were significantly increased (Figure 7C). The top 20 most abundant corona proteins and plasma proteins are presented in Figure 7D. A significant reduction of serum albumin in the relative abundance of captured proteins was observed, indicating that those proteins in different properties competitive for the surface of N4-RMSN_50_@PEG/THPMP during the protein corona process. The detailed proteomic analysis based on the heatmap of the most abundant proteins (minimum relative abundance of 0.5%) is shown in Figure 7E **.** The results suggested a noticeable difference in the profile of the protein content, which was consistent with the results of Figures 7A,B. According to the data of protein corona composition, we further analyzed the possible functional corona proteins of N4-RMSN_50_@PEG/THPMP involved in the biological process of BBB crossing mechanisms. The Venn diagrams showed the number of identified proteins and BBB crossing related proteins (Figure 7F). A total of 273 corona proteins were identified on N4-RMSN_50_@PEG/THPMP. Comparing with the UniProt database, where 85 proteins are currently associated with the transport mechanisms across the BBB; there were three proteins which enriched in the N4-RMSN_50_@PEG/THPMP protein corona. The relative abundances of these three proteins in the protein corona are shown in Figure 7G, verifying the most abundant proteins were as basigin, afamin, and apolipoprotein E (Apo E). Therefore, N4-RMSN_50_@PEG/THPMP mediated BBB transport could be carried out by those transport proteins.

**FIGURE 8:**
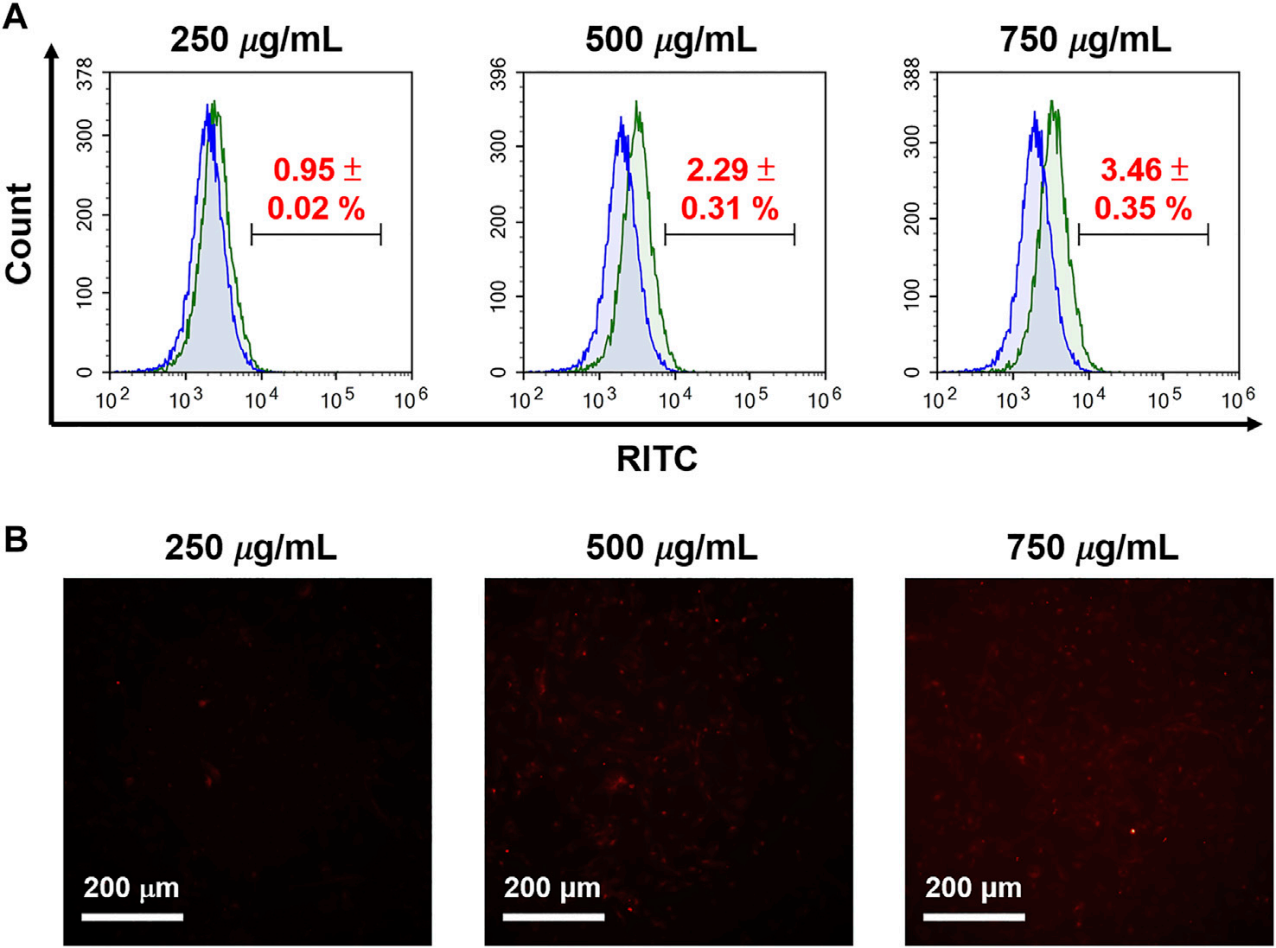
Evaluation of RITC-conjugated N4-MSN_50_@PEG/THPMP for cell uptake. U87-MG cells treated with different concentrations of N4-RMSN_50_@PEG/THPMP (250, 500, and 750 μg/ml) for 24 h. **(A)** Flow cytometry analysis and **(B)** fluorescence microscopy imaging for cell uptake in 10% serum-containing medium. Scale bars: 200 μm.

Furthermore, we explored cell uptake efficacy, focusing on the effects of protein corona and surface modification, proving the internalization of cells and MSNs observed in a 10% FBS-containing medium, which was used to mimic protein corona conditions. It was found that cellular uptake in the human glioma U87-MG cells was not apparent when treated with N4-RMSN_50_@PEG/THPMP, even if at a high concentration (750 μg/ml) based on the results of flow cytometry (Figure 8A) and fluorescence microscope (Figure 8B) detecting the RITC-fluorescence signals from MSNs. N4-RMSN_50_@PEG/THPMS could decrease the cell uptake due to the PEG and negatively charged molecules of the THPMP graft.

## Discussion

The majority of this study is to address the issue of BBB penetration using MSNs. We found only 50 nm of negatively charged MSNs (N2-, N3-, N4-, and N5-RMSN_50_@PEG/THPMP) could be significantly observed outside the blood vessels in the zebrafish brain, meaning MSNs-mediated BBB crossing occurred in charge- and size-dependent manners. Actually, the transport mechanisms based on NPs strategies are mainly through transcytosis by endothelial cells uptake (Tuma and Hubbard, 2003). In addition, NPs induced tight junctions opening or local inflammation of brain cells that promotes the permeabilization of the BBB, allowing substances to cross the BBB temporarily (Yu et al., 2013). There is still much to be explored about the biological process in term of about how and why MSNs could penetrate the BBB. As shown in Figures 2, 4, MSNs with negative zeta potential ranging from −20 mV to −40 mV were essential for crossing the BBB. Even through positively charged Dox loading to let N4-MSN_50_@PEG/THPMP/Dox become more positive, the zeta potential was still in the range of N2-RMSN_50_@PEG/THPMP to N4-RMSN_50_@PEG/THPMP. In addition to charge, factors affecting the efficiency of the substance to partition from blood into the BBB may include tertiary structure, lipid solubility, and protein binding (Banks, 2009). We did not realize the influence of tertiary structure of THPMP molecules to BBB transport. Also, the size of N4-MSN_50_@PEG/THPMP/Dox still maintained stable within the 15 days, implying that the action of N4-MSN_50_@PEG/THPMP/Dox was conducted with no significant aggregation (Figure 5B). Negatively charged MSNs with 200 nm were hardly across the BBB in zebrafish, implying that the size was limited and smaller size was necessary (Figure 4). NPs are smaller than 100 nm in size have good opportunity to efficiently cross the BBB (Betzer et al., 2017). However, NPs engineered through protein–ligand modifications, such as transferrin, albumin, apolipoproteins, and lactoferrin, onto the surface would increase the size up to 1.5-folds (Ku et al., 2010; Zhang et al., 2012; Cui et al., 2013; Hanada et al., 2014). As mentioned earlier, the BBB transport ability by receptor-mediated transcytosis may be interfered when disease progressed due to less expression of protein–ligand receptors (Schenk and De Vries, 2016). Therefore, we here demonstrated negatively charged MSNs without stimuli or ligand/receptor protein functionalization were able to penetrate into BBB *in vivo* through size and charge control and simple surface modification.

Surface properties, such as charge and surface chemistry, are particularly important that influence the biological fate and actions between the NPs and cells (Zhu et al., 2013). There is concern that charge on NPs might induce undesirable biological effects in determining the cytotoxicity and biodistribution (Bhattacharjee et al., 2010; Elci et al., 2016). In our experiments, various types of MSNs on two brain-related cell lines (CTX-TNA2 astrocyte cells and U87-MG glioma cells) by the MTT assay showed no significant change in either concentrations of 100 or 200 μg/ml for 4 or 24 h under 10% serum-containing media condition (Figure 1E), suggesting that different charge MSNs of 50 nm in diameter had no harm and exhibited good biocompatibility *in vitro*. In addition, our previous reports found the strongly positively charged MSNs caused toxicity, leading to 94% of the zebrafish embryos death. On the other hand, no lethality was observed in embryos treated with negatively charged MSNs, which provided valuable insights into the biosafety of better biocompatible nanomaterials before pushing nanoparticles into clinical use (Liu et al., 2015).

NPs presented toxicity issues are associated with their surface functionalization in term of cell inflammatory responses, ROS generation, oxidative stress, and BBB integrity disruption (Trickler et al., 2010; Zhang et al., 2012; Liu et al., 2017). Astrocytes are highly secretory cells and essential for brain homeostasis, which are also known to support the integrity of BBB and responsible to BBB formation and function (Spampinato et al., 2019). Previous study on SiO2 NPs that could disturb the BBB structure and function and induce BBB inflammation through ROS and ROCK-mediated pathways (Liu et al., 2017). The molecular level study proved BBB disruption by SiO2 NPs was attributed to astrocytes-activated VEGF production and the expression of a vascular permeability protein, AQP4, which were both important proteins on BBB integrity. In this study, N4-RMSN50@PEG/THPMP on cytotoxicity evaluation at the molecular level and detailed cellular responses in the CTX-TNA2 astrocyte cell are still unclear. However, our previous study reporting the level of p-p38 was significantly elevated by the positively charged MSNs in RAW 264.7 macrophages, whereas negatively charged MSNs resulted in slight ROS production under serum-containing media condition (Liu et al., 2015), thus proved negatively charged MSNs may be considered as lower cellular toxic.

Over the past 10 years, various types of NPs have been developed as carriers where single or multiple cancer drugs can be delivered with a beautiful and multifunctional design against tumors. The issues of poor drug solubility and stability, non-selectivity to tumor, non-specific cytotoxicity, and high side effects can be successfully improved (Linton et al., 2016; Jahanban-Esfahlan et al., 2018). As a promising nanocarrier, MSNs with unique property of well-ordered mesoporous channel is suitable for drug loading, followed the drug delivery and release. Doxorubicin (Dox) is the most effective chemotherapeutic agent used to treat a broad range of cancers. However, the application of Dox have been severely hindered due to its short half-life, nonspecific distribution, and high cytotoxicity (Yang et al., 2014). Hu X. et al. has demonstrated MSNs with 71.59% of loading efficiency of Dox and cumulative drug release at pH 7.0 and pH 5.0, which is, respectively, about 50 and 67%, could decrease cytotoxicity of Dox in the MCF-7 cells (Hu et al., 2013). As shown in Figures 5A,D, Dox was highly loaded into N4-MSN_50_@PEG/THPMP (78.13% of loading efficiency) and displayed pH-responsive release with a massive release under an acidic condition (pH 5.5). At physiological pH (pH 7.4), N4-MSN_50_@PEG/THPMP/Dox retained more Dox, indicating MSNs may be an opportunity to decrease Dox caused cytotoxicity. As compared to pH 7.4, burst release and higher release of Dox at pH 5.5 could be significantly occurred, which demonstrated that N4-MSN_50_@PEG/THPMP/Dox was capable of being used for brain tumor therapy because tumor microenvironmental is usually acidic (Boedtkjer and Pedersen, 2020). Dox inhibits the progression of topoisomerase II, an enzyme which relaxes supercoils in DNA for transcription in nuclei. Figure 5E showed the cytotoxicity of free Dox was significantly higher than that of N4-MSN_50_@PEG/THPMP/Dox at a concentration of 20 μg/ml, revealing that the slow sustained-release behavior of N4-MSN_50_@PEG/THPMP/Dox might delay the delivery of DOX to nuclei. However, the sustained-release behaviors of N4-MSN_50_@PEG/THPMP/Dox at concentrations of 40 and 60 μg/ml were not significant, which might have been due to the concentration being too high to distinguish. For *in vivo* zebrafish study, it is worth noting that N4-MSN_50_@PEG/THPMP/Dox treatment exhibited a higher survival rate than that of free Dox, which resulted in zebrafish lethal, especially in the treatment with 0.5 mg/ml of Dox (Figure 6). Hence, based on the slow sustained-release of drug, N4-MSN_50_@PEG/THPMP/Dox improved Dox toxicity can be carried out.

Understanding the interactions of serum proteins with NPs surfaces and further manipulating the protein corona of NPs are critical issues in the strategy for effective BBB drug delivery. N4-RMSN_50_@PEG/THPMP has demonstrated the size and charge-dependent ability to cross the BBB. However, potential mechanisms for crossing the BBB are unclear. This study addressed the issue on identifying the corona protein involved in transport across BBB by using LC-MS/MS analysis. As shown in Figure 7A, *in vitro* protein corona analysis by SDS-PAGE revealed the profile difference that reflects the MW and isoelectric point classification differences from protein corona (Figure 7B). Detailed investigation of the relative abundance of the most abundant proteins between the plasma and corona protein can be observed in Figures 7D,E. The aforementioned results suggested that N4-RMSN_50_@PEG/THPMP can selectively capture various kinds of proteins in biofluids. According to LC-MS/MS analysis, three relative abundances (vs mean abundance) of BBB crossing associated proteins were highly related (Figures 7F,G). Basigin is a membrane receptor protein overexpressed in brain endothelial cells, providing receptor-mediated transcytosis delivery to the brain (Zuchero et al., 2016; Christensen et al., 2020). Afamin is a vitamin E-binding glycoprotein contributing to vitamin E transport across the blood–brain barrier through albumin receptors, cubilin, and megalin (Voegele et al., 2002; Kratzer et al., 2009) and is known as well for its neuroprotective effect (Heiser et al., 2002). ApoE, a lipids transporter in the plasma and the central nervous system (Hatters et al., 2006), can promote neurite growth and act as a ligand of lipoproteins receptors for targeting delivery to the brain (Beffert et al., 2004). ApoE is essential for maintaining the integrity of BBB. Here, we proposed that N4-RMSN_50_@PEG/THPMP crossing the BBB is probably attributed to mediate with the three proteins, especially for ApoE.

In addition, previous evidence suggest that surface modification of NPs by introducing the electrical neutral and hydrophilic polymer, polyethylene glycol (PEG), is the most efficient way to decrease the protein adsorption (Partikel et al., 2019), as well as increases the circulating time in the bloodstream and avoid the rapid clearance by the reticuloendothelial system (RES), such as phagocytes in the liver (Kupffer cells) and macrophages and B cells in the spleen (Suk et al., 2016). PEGylation of the NPs proves a decrease in cellular uptake because of the neutral property of PEG that can avoid the cellular interactions between the NPs and the cell surface. PEG conjugation impacts the biodistribution of NPs *in vivo*. In addition to PEG, the charge effect is another critical issue in determining the cellular uptake of NPs. Positively charged NPs are generally beneficial in interacting with the negatively charged cell membrane. In contrast, strongly negatively charged NPs, especially combined with PEGylation, can create steric hindrance and charge barrier, leading to significant inhibition of protein adsorption and less recognition by cells (Figure 8). Lower cellular uptake is helpful to enhance the circulation time by avoiding the clearance by RES that can increase the chance of MSNs crossing the BBB. Here, we proposed the roles of PEGylation and negatively charged molecules THPMP are likely responsible for MSNs mediated BBB crossing through protein corona with transport proteins. This promising and straightforward design may address the challenges and limitations of using NPs to cross the BBB. Concerning brain treatment, negatively charged N4-RMSN_50_@PEG/THPMP of 50 nm size may be an opportunity to improve the outcomes of conventional therapies, thanks to high drug loading capacity, enhanced brain area penetration ability, and slow sustained-release of the drug in acidic sites.

## Conclusion

In conclusion, we demonstrated that MSNs surface modified with molecules of PEG and THPMP could penetrate the BBB without the need for an external stimuli or receptor’s ligand–protein functionalization*.* 50 nm of MSNs. with a critical negative charge ranging from −20 to −40 mV is beneficial to cross the BBB in larval zebrafish, implying that MSNs-mediated BBB penetration acts in charge- and size-dependent manners. As potential drug nanocarriers, the high loading capacity of anticancer drug Dox on MSNs could be achieved, followed by the cumulative time-dependent release, a masking of the cytotoxicity of Dox (at pH 7.4), and massive release (at pH 5.5). With the aforementioned benefits, *in vitro* cytotoxicity and *in vivo* zebrafish toxicity assays showed the decreased cell death and the increased survival rate of zebrafish, respectively. The exciting finding is that three critical proteins were identified, which could be adsorbed onto MSNs, leading to enhanced BBB penetration. The study offers fundamental knowledge of the success of NPs-enabled BBB crossing and valuable insigne for future applications in brain disease therapy.

## Data Availability

The datasets presented in this study can be found in online repositories. The names of the repository/repositories and accession number(s) can be found at: https://www.ebi.ac.uk/pride/archive/, PXD033783.
