# Supplementary material for: Bridging Size and Charge Effects of Mesoporous Silica Nanoparticles for Crossing the Blood–Brain Barrier
Source: Front Chem. 2022 Jun 27;10:931584. doi: 10.3389/fchem.2022.931584 (PMC9307501; doi:10.3389/fchem.2022.931584)
Supplement: Supplementary file 1 [file Presentation1.PPTX]

## Slide 1
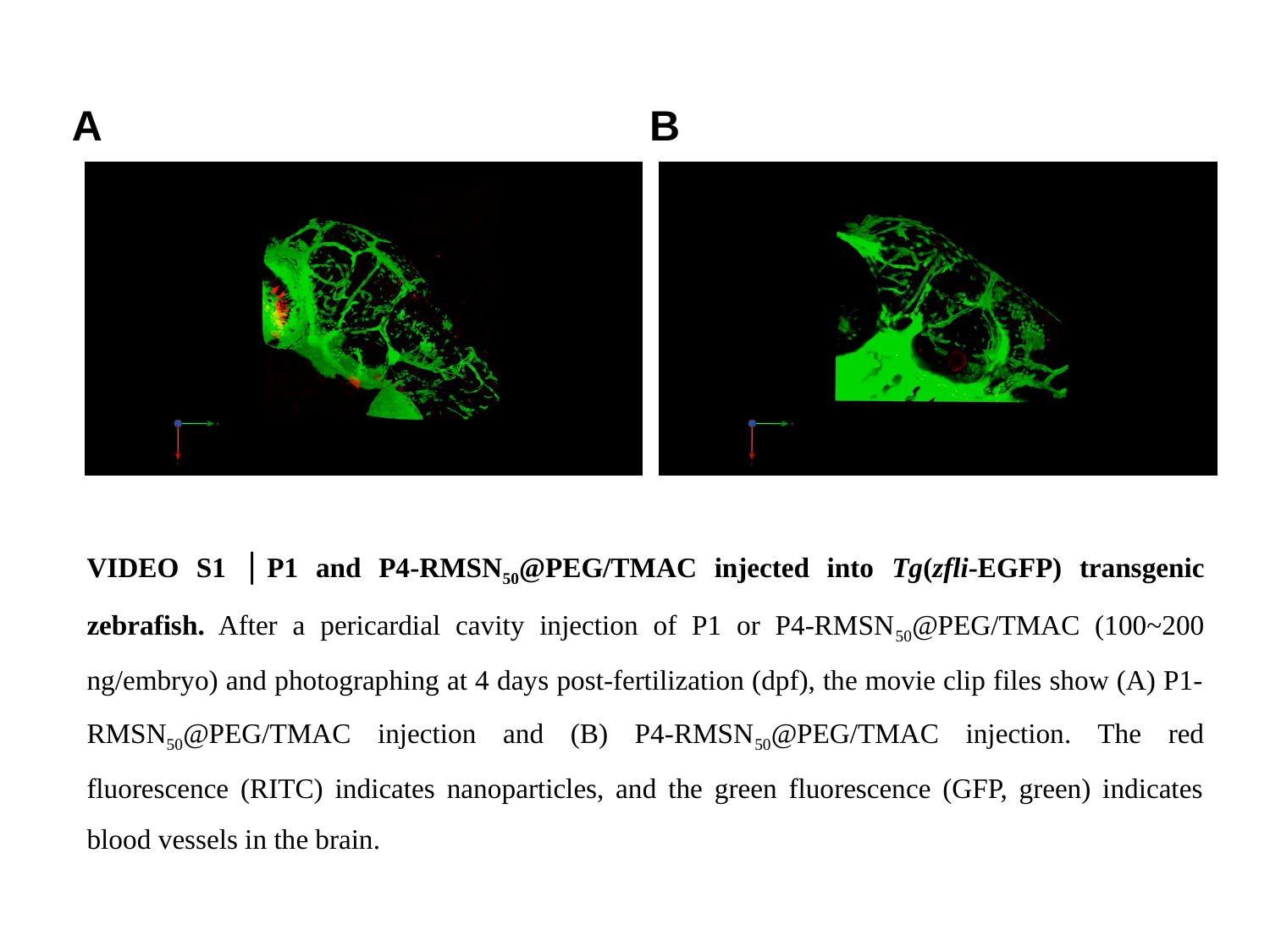

B
A
VIDEO S1 │ P1 and P4-RMSN50@PEG/TMAC injected into Tg(zfli-EGFP) transgenic zebrafish. After a pericardial cavity injection of P1 or P4-RMSN50@PEG/TMAC (100~200 ng/embryo) and photographing at 4 days post-fertilization (dpf), the movie clip files show (A) P1-RMSN50@PEG/TMAC injection and (B) P4-RMSN50@PEG/TMAC injection. The red fluorescence (RITC) indicates nanoparticles, and the green fluorescence (GFP, green) indicates blood vessels in the brain.
