# Supplementary material for: Bridging Size and Charge Effects of Mesoporous Silica Nanoparticles for Crossing the Blood–Brain Barrier
Source: Front Chem. 2022 Jun 27;10:931584. doi: 10.3389/fchem.2022.931584 (PMC9307501; doi:10.3389/fchem.2022.931584)
Supplement: Supplementary file 2 [file Presentation3.PPTX]

## Slide 1
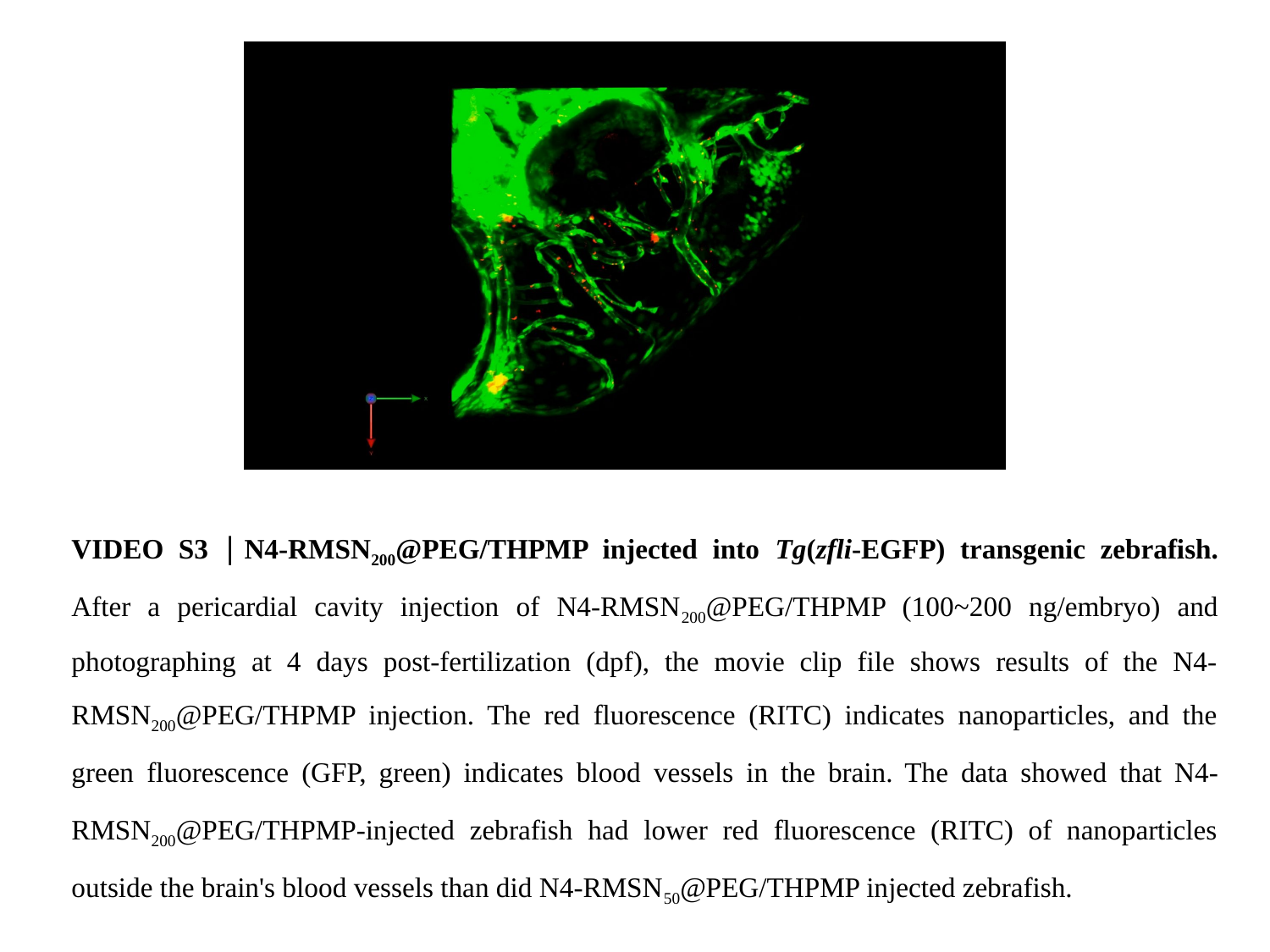

VIDEO S3 │ N4-RMSN200@PEG/THPMP injected into Tg(zfli-EGFP) transgenic zebrafish. After a pericardial cavity injection of N4-RMSN200@PEG/THPMP (100~200 ng/embryo) and photographing at 4 days post-fertilization (dpf), the movie clip file shows results of the N4-RMSN200@PEG/THPMP injection. The red fluorescence (RITC) indicates nanoparticles, and the green fluorescence (GFP, green) indicates blood vessels in the brain. The data showed that N4-RMSN200@PEG/THPMP-injected zebrafish had lower red fluorescence (RITC) of nanoparticles outside the brain's blood vessels than did N4-RMSN50@PEG/THPMP injected zebrafish.
