# Supplementary material for: Bridging Size and Charge Effects of Mesoporous Silica Nanoparticles for Crossing the Blood–Brain Barrier
Source: Front Chem. 2022 Jun 27;10:931584. doi: 10.3389/fchem.2022.931584 (PMC9307501; doi:10.3389/fchem.2022.931584)

Supplementary Material

**Bridging Size and Charge Effects of Mesoporous Silica Nanoparticles for Crossing the Blood-Brain Barrier**

Yi-Ping Chen^1,2†^, Chih-Ming Chou^3,4†^, Tsu-Yuan Chang^3,4^, Hao Ting^3,4^, Julien Dembélé^5^, You-Tai Chu^1^, Tsang-Pai Liu^6^, Chun A. Changou^7^, Chien-Wei Liu^8^ and Chien-Tsu Chen^3,4*^

^1^Graduate Institute of Nanomedicine and Medical Engineering, College of Biomedical Engineering, Taipei Medical University, Taipei, Taiwan, ^2^International PhD Program in Biomedical Engineering, College of Biomedical Engineering, Taipei Medical University, Taipei, Taiwan, ^3^Graduate Institute of Medical Sciences, College of Medicine, Taipei Medical University, Taipei, Taiwan, ^4^Department of Biochemistry and Molecular Cell Biology, College of Medicine, Taipei Medical University, Taipei, Taiwan, ^5^Graduate Institute of Biomedical Materials and Tissue Engineering, College of Biomedical Engineering, Taipei Medical University, Taipei, Taiwan, ^6^Department of Surgery, Mackay Memorial Hospital, Taipei, Taiwan, 7The PhD Program for Translational Medicine, College of Medical Science and Technology, Taipei Medical University, Taipei, Taiwan, 8Department of Information Management, St. Mary’s Junior College of Medicine, Nursing and Management, Yilan, Taiwan

**†These authors have contributed equally to this work**

*** Correspondence:**Chien-Tsu Chen
chenctsu@tmu.edu.tw

**FIGURE**


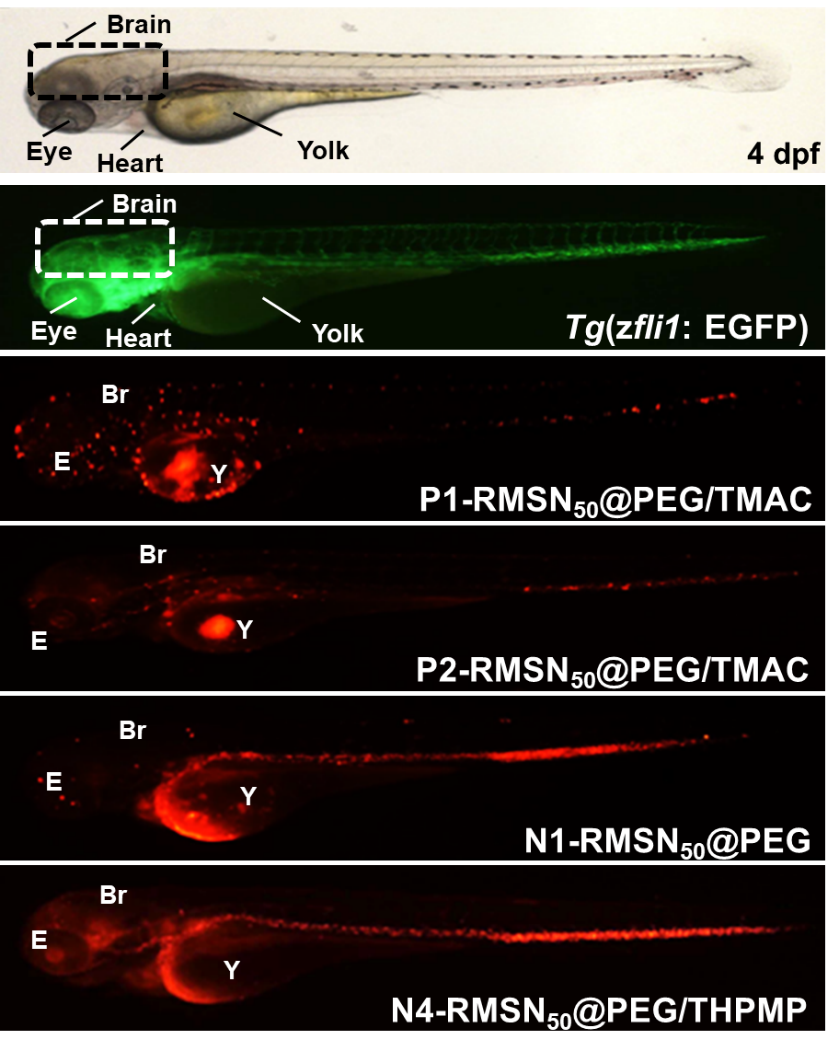


**FIGURE S1****│**Fluorescence microscopic images of the distribution of various types of RITC-conjugated mesoporous silica nanoparticles (RMSNs) in the larval zebrafish brain. Br, brain; E, eye; Y, yolk.

**TABLES**

**TABLE S1│**Summary of hydrodynamic diameter distributions and zeta potential of various types of RITC-conjugated mesoporous silica nanoparticles (RMSNs) in different solutions.

**
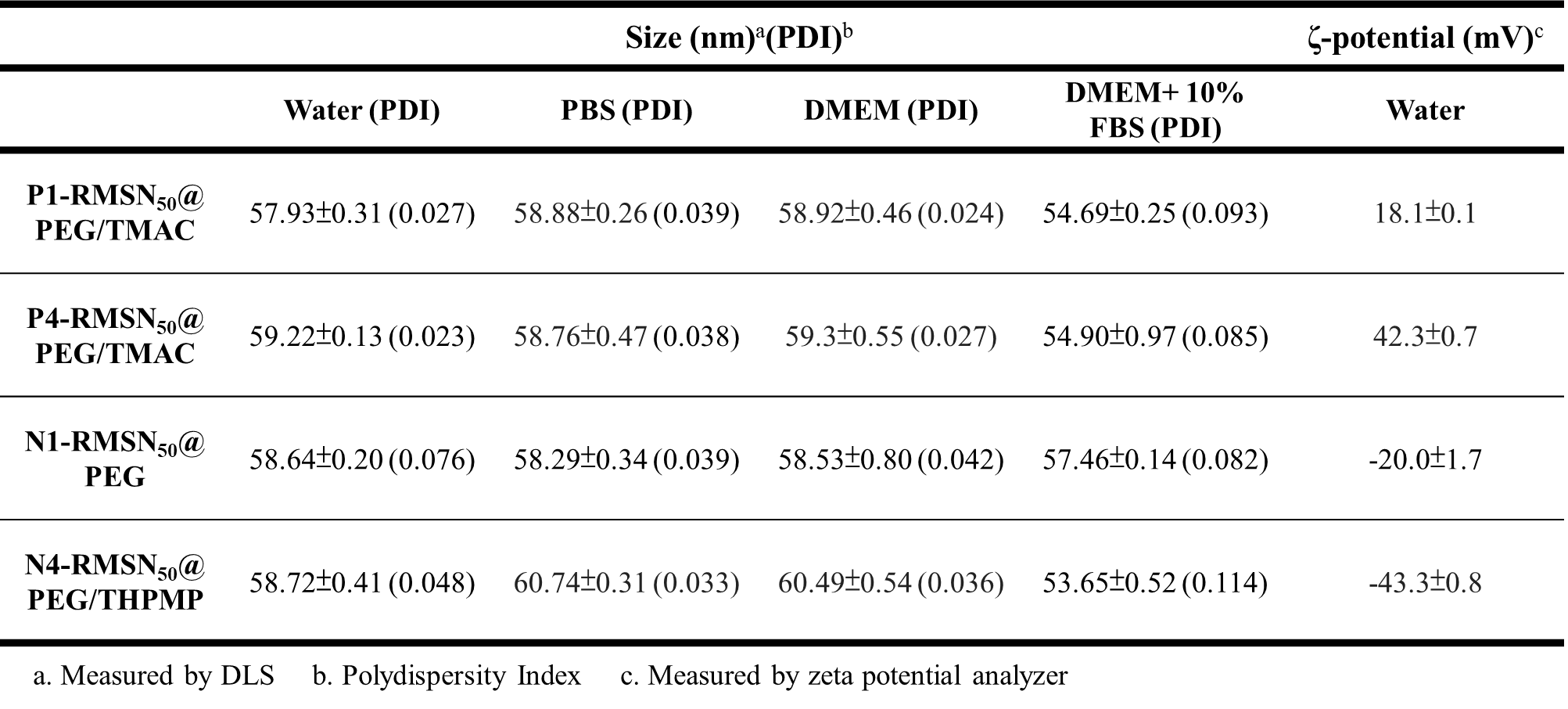
**

**TABLE S2│**Summary of hydrodynamic diameter distributions and zeta potential of various types of RITC-conjugated mesoporous silica nanoparticles (RMSNs) in different solutions.


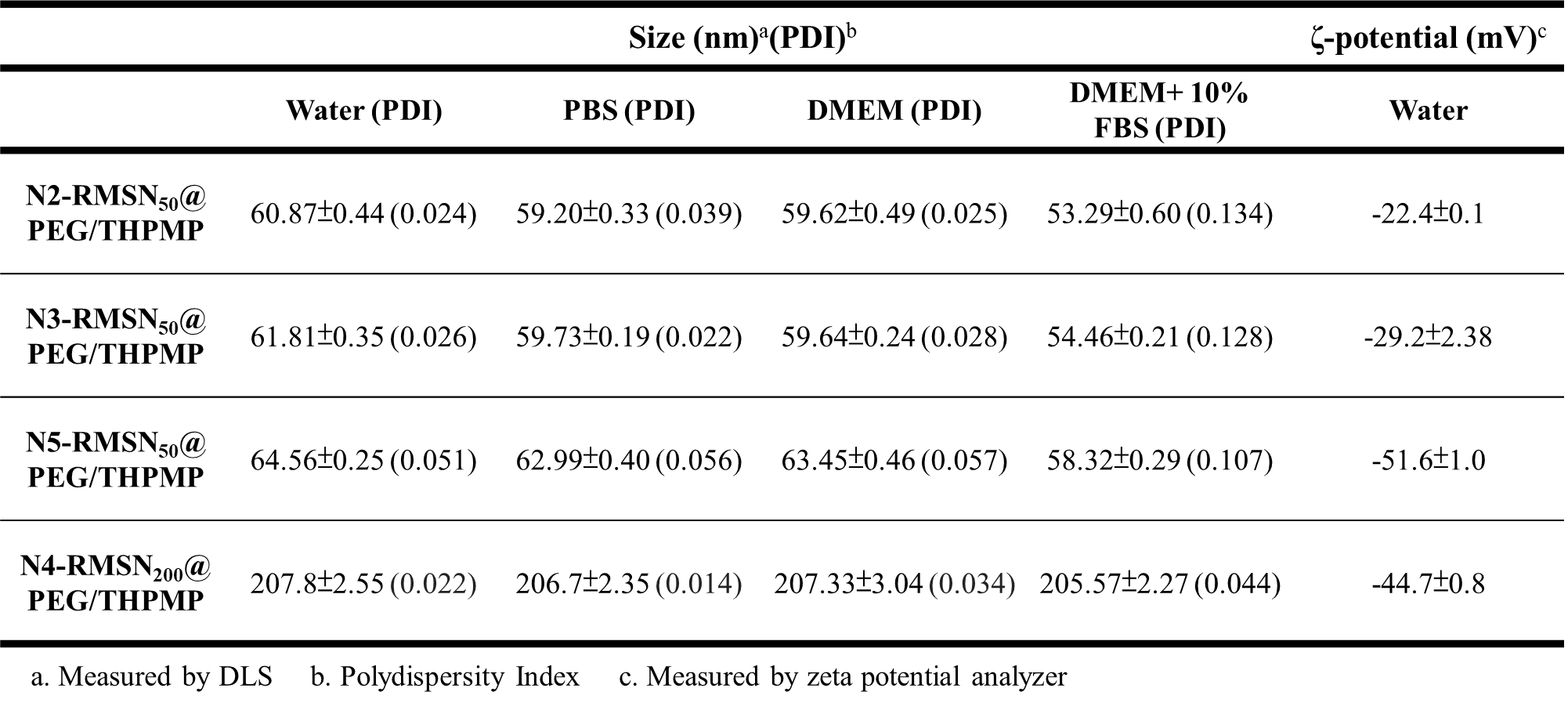


**TABLE S3│**Summary of survival rate of zebrafish with different treatments.


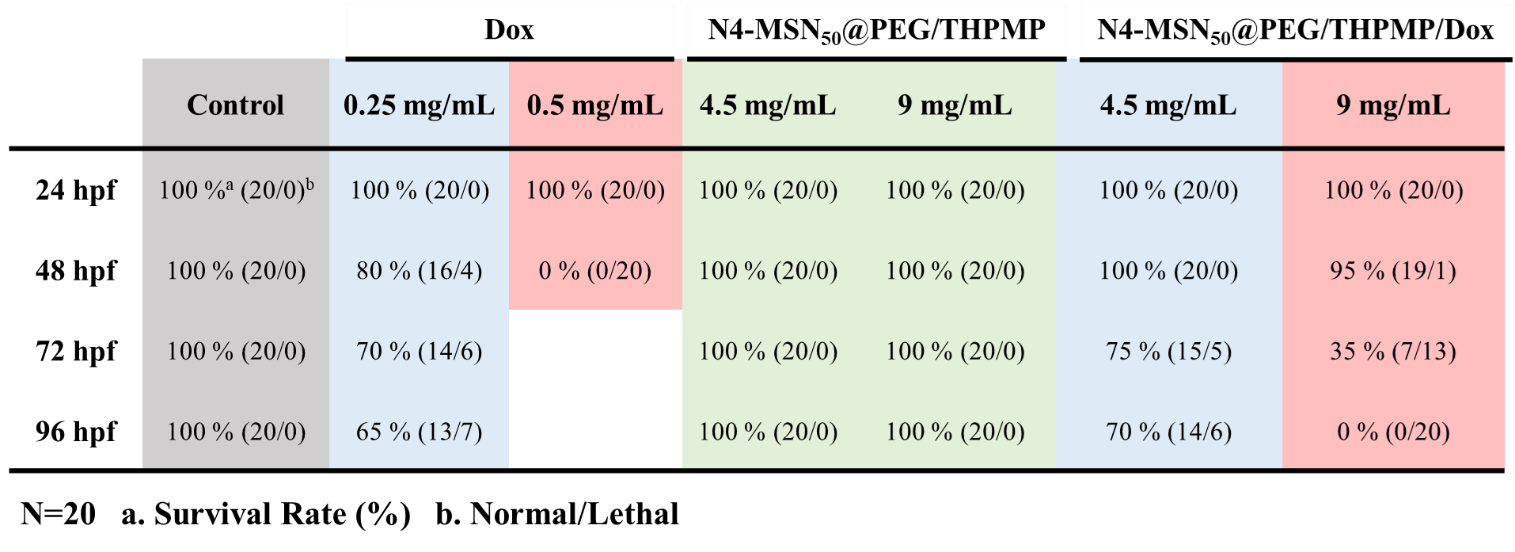

Supplement: Supplementary file 4 [file DataSheet1.docx]
